# Regenerative Glycopeptide Scaffolds Enhance BMP-4 Activity To Treat Pediatric Glioma

**DOI:** 10.1007/s40883-025-00543-5

**Published:** 2025-12-19

**Authors:** Cara S. Smith, Timmy Fyrner, Nicholas A. Sather, Mark T. McClendon, Oscar A. Carballo-Molina, Charles D. James, Tadanori Tomita, Guifa Xi, Samuel I. Stupp

**Affiliations:** 1 Department of Biomedical Engineering, Northwestern University, Evanston, IL 60208 USA; 2 Center for Regenerative Nanomedicine, Northwestern University, Chicago, IL 60611 USA; 3 Department of Chemistry, Northwestern University, Evanston, IL 60208 USA; 4 Division of Pediatric Neurosurgery, Ann & Robert H. Lurie Children’s Hospital, Northwestern University Feinberg School of Medicine, Chicago, IL 60611 USA; 5 Department of Neurological Surgery, Northwestern University Feinberg School of Medicine, Chicago, IL 60611 USA; 6 Department of Materials Science and Engineering, Department of Chemistry, Northwestern University, Evanston, IL 60208 USA; 7 Department of Medicine, Northwestern University, Chicago, IL 60611 USA

**Keywords:** Peptide amphiphiles, Sulfated glycopeptide, Glycochemistry, Bone morphogenetic protein 4, Pediatric high-grade gliomas, Sulfated glycosaminoglycan mimetic

## Abstract

**Abstract:**

Pediatric high-grade gliomas (pHGGs) are among the most devastating cancers in children. These tumors have remained largely incurable, despite the many approaches that have been applied for their treatment. Here we use scaffolds of glycopeptide nanostructures designed for regenerative therapies to bind and present bone morphogenetic protein (BMP-4) in vivo to differentiate glioma cells and render them more susceptible to traditional chemotherapeutics. Interestingly, we discovered that the presentation of BMP-4 on these glycopeptide structures alone, without the use of a traditional chemotherapy, resulted in reduced tumor growth and enhanced survival in an orthotopic xenograft pediatric high-grade glioma tumor mouse model. Thus, this strategy has the potential to serve as a future chemotherapy-free platform for treating pHGGs which may have significantly reduced comorbidities.

**Lay Summary:**

Pediatric glioblastoma (pGBM) is an aggressive brain cancer with poor survival rates despite surgery, radiation, and chemotherapy. The growth factor BMP-4 shows promise as a treatment, but its short half-life limits its potential as a future pGBM therapeutic. We developed nanostructures made from sugar-inspired molecules known as glycopeptide amphiphile molecules (gPA) that can bind, stabilize, and enhance the activity of BMP-4. When presented on gPA, BMP-4 directed pGBM cells to become less stem cell-like which slowed tumor growth in a mouse model. This approach highlights a potential strategy to improve BMP-4 delivery to advance therapeutic options for children with pGBM.

**Future Work:**

Development of chemically scalable glycosylated supramolecular structures, such as the one described here, can be used to bind and present BMP-4 as well as other proteins for future drug delivery applications in the nervous system and beyond. Future research should also investigate how these therapies can be co-administered with chemotherapeutics.

**Graphical Abstract:**

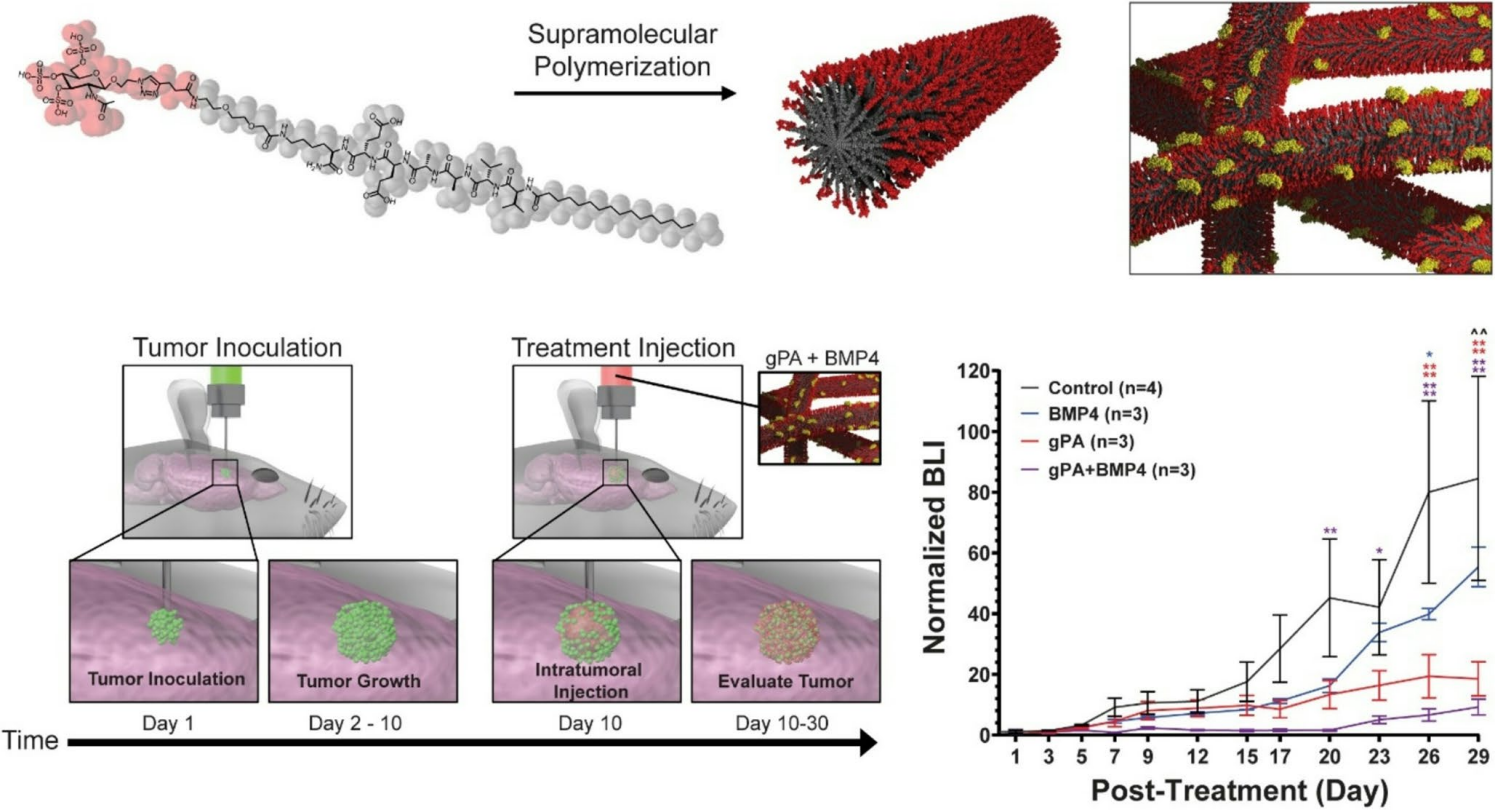

**Supplementary Information:**

The online version contains supplementary material available at 10.1007/s40883-025-00543-5.

## Introduction

Pediatric high-grade gliomas (pHGGs) are among the most devastating cancers that occur during childhood [1]. While most patients will receive aggressive surgical resection in combination with radiation and chemotherapy, the five-year survival rate of children with pHGG is 28.1% [2], and for tumors that recur, the expected length of survival is approximately 5.6 months [3]. Genetic and epigenetic variations in pHGGs, which differ from their adult counterparts, add difficulties in pursuing novel, targeted therapies [4–6]. 

Bone morphogenetic protein 4 (BMP-4), a critical protein in embryonic and central nervous system (CNS) development, has been implicated as both a potential prognostic factor and therapeutic treatment for HGG [7–12]. Specifically, BMP-4 has been shown to inhibit the proliferation of glioblastoma (GBM) cells [11], encourage the differentiation of brain tumor-initiating precursor cells (BTICs) into non-stem-like neural lineages [8, 13], and reduce the overall level of multidrug resistance (MDR) of tumors [9]. Unfortunately, clinical translation of BMP-4 as a pHGG treatment is complicated by the protein’s short half-life in vivo, requiring high and frequent doses of the protein to maintain efficacy as demonstrated by a recent Phase I human clinical trial (ClinicalTrials.gov Identifier: NCT02869243) utilizing convection-enhanced delivery (CED) to study the efficacy of multiple increasing doses of BMP-4 [14]. Other BMP-4 delivery strategies, such as stimulating local cells to release BMP-4 via viral vector delivery [15] or delivering exogenous cells that release BMP-4 [10, 16–18], can eliminate the need for multiple invasive injections. However these methods do not address the short half-life for BMP-4 once it is released from the cells.

One strategy to improve the stability and therapeutic efficacy of proteins such as BMP-4 is to stabilize and present them on the surface of carriers such as polymeric scaffolds [19] or nanoparticles [20]. Peptide amphiphiles (PAs), which are comprised of an aliphatic tail covalently coupled to peptide sequences, have the ability to self-assemble in aqueous conditions into 1D nanostructures which form hydrogels in the presence of electrolytes [21–24] that can be designed to bind specifically to bioactive biopolymers such as heparin [25] and growth factors such as BMP-2 [26, 27] and TGFβ−1 [28, 29]. Recently, a sulfated glycopeptide PA (gPA) developed by the Stupp laboratory has shown promise in its ability to non-covalently bind several different proteins, including BMP-4, thus extending their bioactive half-life in vitro and in vivo [24]. The gPA molecular design was inspired by the highly sulfated glycosaminoglycans (GAGs) heparan sulfate and heparin, which interact with an array of different proteins that contain heparan sulfate binding domains [30]. In the case of BMP-4, the heparan sulfate binding domain corresponds to the peptide sequence 1-KKNKNCRRH-9 near the N-terminus, where both BMP-2 and BMP-4 exhibit typical Cardin–Weintraub (CW) motifs, with XBBXBX and XBBBXBX arrangements, respectively [31]. The main feature of the gPA molecule is a chemically synthesized N-terminal tri-sulfated monosaccharide, which is displayed on the surfaces of supramolecular nanofibers formed by the gPA. This synthetically derived sulfated supramolecular GAG mimetic mimics the structure of heparan sulfate and heparin while offering additional clinical advantages. For example, the relatively short half-life of heparan sulfate and heparin (t_1/2_ ~30 min to 7 h) [32] poses a major limitation for their clinical translation in the context of BMP-4 delivery for pHGG treatment. Additionally, while heparan sulfate and heparin are known to cause anti-clotting effects due to the inhibition of factor Xa [33, 34], we showed that gPA did not inhibit factor Xa, reducing the risk of hemorrhaging. However, while this previous work demonstrated effective binding of the gPA to BMP-4 using surface plasmon resonance (SPR) spectroscopy, in vitro and in vivo studies investigating binding mechanism and bioactive efficacy were focused solely on BMP-2 [24]. 

There has been great interest over the past few decades in understanding the pivotal role of glycans in biological systems, highlighting the expansive potential of translational biology in advancing biomedical research [35–38]. Despite significant synthetic advancements [39, 40], challenges persist in the development of therapies that authentically replicate the intricate biological complexity of glycan assemblies. Additionally, creating therapies capable of controlling and sustaining functionality across diverse biomedical applications remains a formidable task [41]. Our previous work has demonstrated the utility of PAs as a versatile platform for effectivity presenting various biological cues, including sulfated glycans [24]. In this context, we have explored a scalable synthetic route, and also investigated shelf-life stability for both the sulfated azido-glycans (4) (Scheme S1) and the gPA over the past few years (see figures S30-S32), including multiple freeze-thaw cycles, which enhance the translational potential of gPAs as future supramolecular therapeutics.

In this work, we investigate if gPA nanostructures and the hydrogels they form can enhance the therapeutic effect of BMP-4 for glioblastoma on pHGG cells both in vitro and in vivo. Experiments were first directed to establish the bioactivity of BMP-4 bound to gPA nanostructures in vitro. Next, we evaluated the ability of the gPAs to reduce tumor growth and extend animal survival in an orthotopic pHGG xenograft mouse model. Finally, in order to assess the clinical potential of the regenerative biomaterial to treat glioblastoma we investigated its role at modulating chemotherapy treatments.

## Results and Discussion

**Fig. 1:**
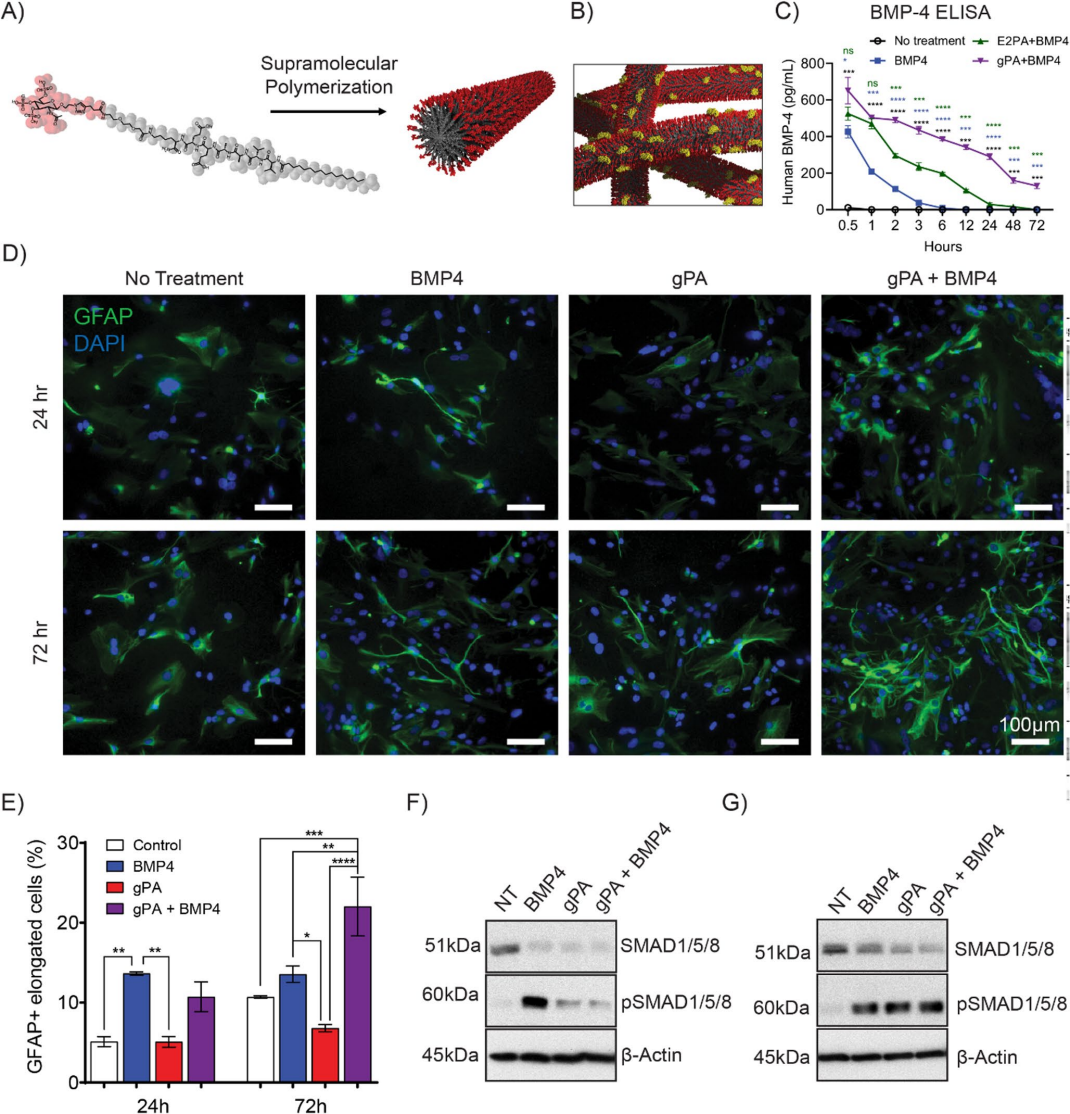
**BMP-4 bound to gPA nanostructures differentiates KNS42 cells and activates the BMP-4 receptor pathway **(**A**) The gPA molecular structure is comprised of a PA molecule (dark grey) covalently attached to a tri-sulfated monosaccharide group (red). gPA self-assembles in solution into high-aspect ratio supramolecular polymer fibers. (**B)** gPA fibers bind and protect BMP-4 protein (yellow) through interactations between BMP-4’s heparan sulfate binding domain and the gPA’s tri-sulfated monosaccharide group (red). (**C**) The BMP-4 ELISA illustrates that gPA stabilizes BMP-4 (purple) and increases its half-life by interacting with the heparan sulfate–binding domain, in comparison with BMP-4 alone (blue) or E2-PA (green). The concentration of BMP-4 was determined at 0.5, 1, 2, 3, 6, 12, 24, 48, and 72 h. (**D**) Representative micrographs of KNS42 cells treated in solution for 24 and 72 h. Cells were stained for glial fibrillary acidic protein (GFAP) (green) and DAPI (blue). (**E**) Quantification of the percentage of GFAP positive elongated cells present in culture 24 and 72 h after treatment. Western blot of SMAD1/5/8, pSMAD1/5/8, and β-actin in KNS42 cells treated with recombinant BMP-4 (5 ng/mL), gPA (25 µM), or a combination of gPA (25 µM) and BMP-4 (5 ng/mL) for (F) 1 h and (**G**) 6 h. For (**E**) a two-way ANOVA with mulitple comparisons (α = 0.05) was performed: (*) *P* < 0.05, (**) *P* < 0.01, (***) *P* < 0.001, (****) *P* < 0.0001

We sought to improve BMP-4’s therapeutic efficacy, given limitations on its use due to its short half-life in vitro and in vivo [42–44]. To accomplish this goal, we investigated the binding of BMP-4 to a chemically synthesized, xeno-free gPA previously developed for regenerative scaffolds, designed to mimic both the structure and protein binding capacity of heparan sulfate. Heparan sulfate is a highly sulfated glycosaminoglycan known for its ability to bind proteins and optimize their presentation to cell receptors through the so called “heparan sulfate binding domains” in many growth factors [30]. To utilize glycated biologics in biomedical applications, such as the gPA [24], it is critical to obtain well-characterized product in substantial quantities. Thus we initiated this work by enhancing our synthetic route to gPA by minimizing the total number of chemical transformations, starting from a readily available commercial precursor. In addition, the purification process has been streamlined, minimizing the reliance on column chromatography, and instead utilizing crystallizations for purifications (Scheme S1). The backbone of the gPA structure is V_2_A_2_E_2_, a previously studied PA referred to as E2-PA based on the presence of two glutamic acid residues in the sequence, which forms stable high-aspect ratio nanofibers in solution (Fig. 1A). To enhance accessibility of the protein-binding moiety, an oligo(ethylene glycol) linker and lysine residue were inserted between a terminal glutamic acid and the tri-sulfated monosaccharide moiety (2-acetamido-3,4,6-tri-O-sulfo-2-deoxy-β-D-glucopyranoside) (Fig. 1A) [24]. An oligo(ethylene glycol) linker, inserted between the last glutamic acid and the tri-sulfated monosaccharide group, is utilized to further promote accessibility to the protein-binding portion of the structure (Fig. 1A) [45]. Previous surface plasmon resonance (SPR) experiments have indicated that gPA is capable of specifically binding BMP-4 to the nanofiber surface (Fig. 1B) via its heparin binding domain [24, 46]. The concentration of BMP-4 was quantified using an enzyme-linked immunosorbent assay (ELISA) (Fig. 1C) at each time point (0.5, 1, 2, 3, 6, 12, 24, and 72 h) to assess how gPA extends the half-life of BMP-4. Here, E2-PA served as a control to demonstrate the importance of the sulfated glycan epitope on gPA in optimizing its stabilizing interactions with the heparan sulfate–binding domain of BMP-4, and after 2 h, gPA exhibited significantly greater stabilization of BMP-4 than E2-PA. In the orthotopic tumor model, gPA was loaded with BMP-4 at a level representing less than 1% of its binding capacity, indicating substantial additional loading potential without the safety concerns typically associated with heparin or heparan sulfate (see supporting information for calculations).

Because prior studies have demonstrated the ability of BMP-4 to differentiate stem-like glioma precursor cells into a non-stem-like phenotype [8, 13], we next evaluated the ability of gPA bound BMP-4 to alter pHGG cell phenotype in vitro using the human glioma cell line KNS42. Specifically, we assessed the ability of recombinant BMP-4 and gPA bound BMP-4 to differentiate glioma cells into elongated astrocytes that are positive for the marker glial fibrillary acidic protein (GFAP), indicating a shift into a non-stem-like, non-cancerous phenotype. We also measured the extent of cell proliferation after 24 and 72 h of treatment, as non-stem like phenotypes will have slower proliferation than the stem-like glioma phenotype (Fig. 1D-E, S1A-B). After only 24 h, KNS42 cells treated with gPA bound BMP-4 had a slight increase in the number of GFAP positive cells when compared to cells treated with gPA alone or those used as a control without any treatment, while recombinant BMP-4 showed a significantly higher number. However, after 72 h, BMP-4 was significantly less efficient in this differentiation process than gPA bound BMP-4, which was significantly more effective than all other treatments (Fig. 1D-E). This effect is attributed to the short half-life of free BMP-4 compared to bound BMP-4. We also noted differences in proliferation of KNS42 cells, with a significant decrease in cell proliferation for gPA bound BMP-4 treated cells, another potential indicator of a shift to a more non-stem-like phenotype (Figure S1A-B). After 72 h, gPA bound BMP-4 treatment led to a lower number of cells as well as a lower number of Ki67 + cells, a marker for cell proliferation. (Figure S1A-B). We next examined whether PA bound BMP-4 was able to maintain its ability to interact with and activate BMP-4 receptors expressed on the surface of KNS42 cells. Using Western blot we were able to establish an increased expression of phosphorylated SMAD1/5/8 (pSMAD1/5/8) in a time dependent manner, an indicator of BMP-4 pathway activation following treatments of recombinant BMP-4 and gPA bound BMP-4 (Fig. 1F-G). After only 1 h of treatment, recombinant BMP-4 had the strongest upregulation of pSMAD1/5/8, though both gPA and the gPA bound BMP-4 achieved some level of upregulation (Fig. 1F). However, while recombinant BMP-4 still showed some level of pSMAD1/5/8 upregulation after 6 h of treatment, gPA bound BMP-4 showed the highest levels of pSMAD1/5/8 expression after this period of time (Fig. 1G). While we also observed upregulation of pSMAD1/5/8 for gPA only treated cells, we attribute this to the nanostructures binding endogenous BMP-4 or other proteins. These differences are attributed to the ability of the gPA to non-covalently bind BMP-4. To determine if we would see a similar trend in a different pediatric glioblastoma cell line, we repeated the experiment in CHLA200 cells (Figure S1C-D). Interestingly, we found that both BMP-4 alone and gPA bound BMP-4 similarly upregulated pSMAD1/5/8 in comparison to a no treatment control. We attribute these differences to established genetic variations between pediatric glioma cell lines [47] which likely cause them to differentially respond to external signaling factors, such as BMP-4. In both of these studies, we observed no pSMAD1/5/8 upregulation in our no treatment control groups and some pSMAD1/5/8 upregulation in our BMP-4 only treated groups, indicating that changes in pSMAD1/5/8 levels across experimental conditions can likely be attributed to the addition of BMP-4. Furthermore, this experimental design reflects prior work which similarly probed the ability of BMP-4 to regulate pSMAD1/5/8 activation [48, 49]. 

Once we confirmed the ability of gPA bound BMP-4 to influence pHGG cell behavior in vitro, we next wanted to evaluate the clinical relevancy of this therapeutic treatment in vivo. Because the gPA has previously only been studied in vivo in the context of sequestering BMP-2 for bone regeneration in the spine [24], we first wanted to confirm whether intertumoral delivery of gPA bound BMP-4 for pHGG treatment was feasible. Specifically, we wanted to evaluate the gPA’s mechanical properties, which could help us establish its injectability, and investigate whether gPA could successfully diffuse through brain tissue to cover the entire area of the tumor. To accomplish this, we investigated both the rheological properties of gPA as well as the intracranial distribution of gPA administered directly into mouse cortices via a stereotactic injection, mimicking how the therapy would be injected in an in vivo model of CNS tumor treatment. As a control, we also evaluated nanofibers comprised of E_2_-PA (C_16_V_2_A_2_E_2_) molecules, which contain the non-bioactive backbone of the gPA molecules (without the sulfated monosaccharide group) and are well-established to form nanofibers and gel in the presence of divalent cations (Figure S2A-B), and nanofibers comprised of a 50:50 molar ratio mixture of gPA: E_2_-PA (Mix-PA) (Figure S2C) [50–52]. We first measured the viscosity and gel modulus of gPA, E_2_-PA, and Mix-PA in a cerebrospinal fluid (CSF) mimetic solution (2.5mM CaCl_2_, 150mM NaCl) (Figure S2D-E). The E_2_-PA, which has exposed glutamic acids on its nanofiber surface, had both a significantly higher viscosity and gel modulus than both the gPA and Mix-PA. While the gPA and Mix-PA had a similar lower viscosity, the gPA had a significantly lower gel modulus than Mix-PA. We hypothesize that the differences in viscosity are likely due to the differences in charge density on the nanofiber surfaces, as we have previously reported that gPA structures were generally more negative than E2 PA structures via Zeta potential measurements [24]. The pendant tri-sulfated saccharide groups on gPA have significantly higher charge than E_2_-PA [24] and we have previously discovered that higher charge density on nanofiber surfaces results in lower viscosity due to a reduction in inter-fiber interactions [53, 54]. In comparison, the gel modulus in artificial CSF is determined by the ability of the nanofibers to ionically crosslink via divalent Ca^2+^ ions. The glutamic acids on the E_2_-PA surface are stronger chelators of Ca^2+^ than the sulfate groups on gPA due to the carboxylates [55], which explains why E_2_-PA has the highest gel modulus, followed by Mix-PA (which has 50% of the glutamate residues compared to E_2_-PA). Taken together, the low viscosity and gel modulus suggest that the gPA is a good candidate molecule to diffuse easily through the brain parenchyma and fully covering the tumor tissue.

Next, we sought to determine whether the rheological differences among gPA, E_2_-PA, and Mix-PA would correlate to differences in diffusion through brain tissue following stereotactic injection into mouse cortices. To visualize the distribution of material throughout the brain tissue, we mixed a 1 mol% of Cy3-labelled E_2_-PA with the gPA, E_2_-PA, and Mix-PA to create fluorescently labeled nanostructures. As an additional control, we also considered the distribution of free Cy3 monomers dissolved in saline. While free Cy3 diffused freely through the brain tissue, gPA, E_2_-PA, and Mix-PA were found to be more concentrated around the site of injection both at ½ hour and 1 h after treatment (Figure S2F). When comparing between the different PA structures, the gPA appeared to have the greatest area of distribution throughout the brain tissue while the E_2_-PA and Mix-PA appeared more concentrated at the site of injection. This outcome correlates with both the viscosity and gel modulus results – the gPA, which has a lower viscosity and gel modulus, is able to more easily spread through brain tissue while E_2_-PA and Mix-PA, which have greater capacity to gel, are localized more closely at the injection site. From these experiments, we established that the gPA is not only able to be successfully injected into the brain, but is also able to easily diffuse through brain tissue, increasing the likelihood of targeting a greater volume of pHGG.

**Fig. 2:**
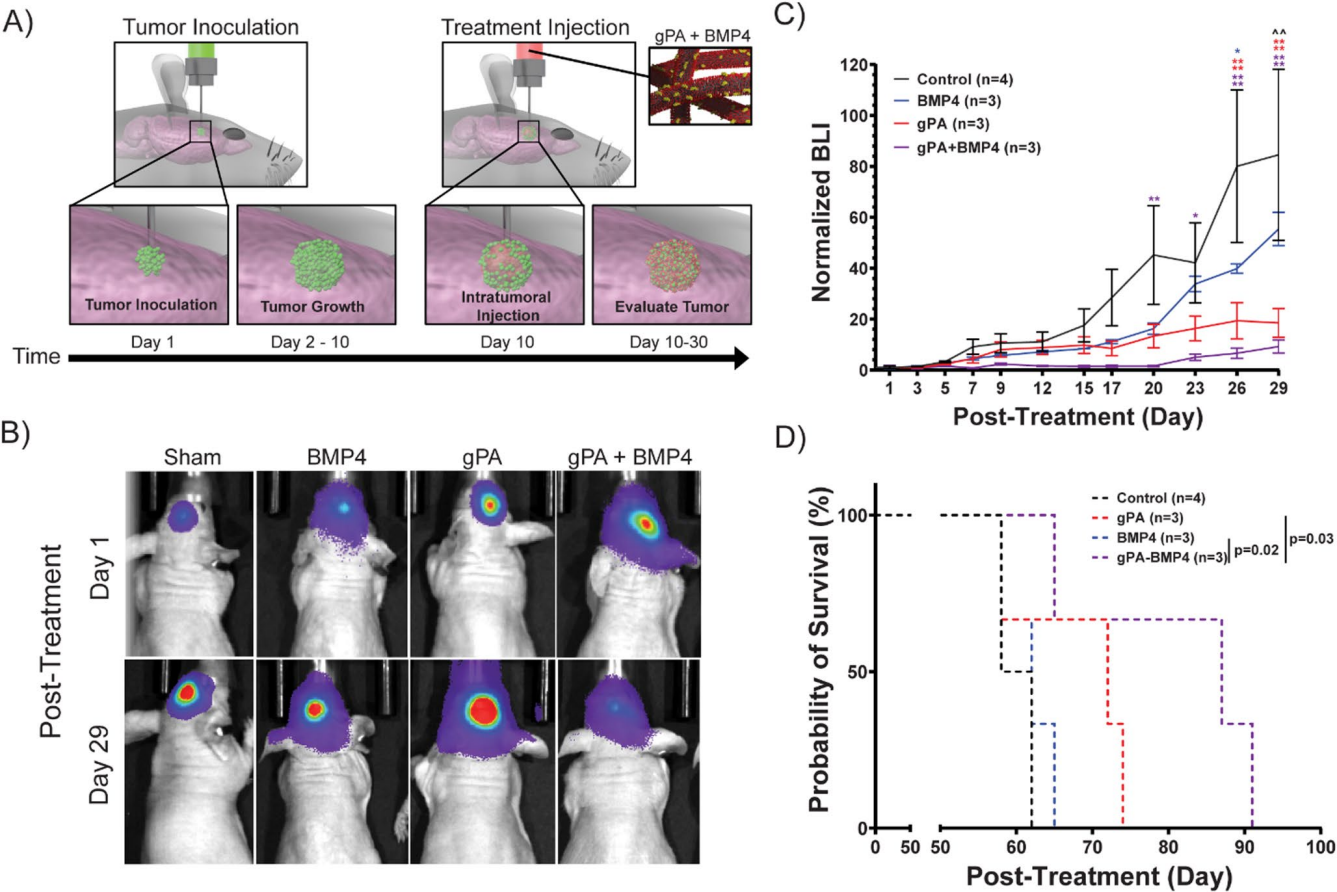
**gPA bound BMP-4 reduces tumor growth and improves survival in an orthotopic xenograft intracranial glioblastoma tumor model. (A)** Schematic of in vivo orthotopic xenograft intracranial glioblastoma tumor model. Nude mice were first inoculated with KNS42 cells transfected with luciferase. After 10 days of tumor growth, treatments (saline, BMP4, gPA, or gPA + BMP4) were injected at the tumor site. Tumor growth was then monitored over the course of 29 days with bioluminescence imaging (BLI). **(B)** Representative micrographs of BLI imaging at 1 and 29 days post-treatment. **(C)** Normalized BLI **(D)** Probability of survival over the course of 100 days. For **(C)** a two-way ANOVA with muliple comparisions (α = 0.05) was performed: (*) *P* < 0.05, (**) *P* < 0.01, (***) *P* < 0.001, (****) *P* < 0.0001. For **(D)** a log-rank (Mantel-Cox) test was performed

Once we established that the gPA could be successfully administered via stereotactic injection, we used an in vivo orthotopic xenograft pHGG mouse model to study the efficacy of gPA bound BMP-4 in reducing tumor size and extending survival over time (Fig. 2A). Nude mice were inoculated with luciferase transfected KNS42 cells by stereotactic injection into the mouse cortex. After 10 days, treatments were administered in a final volume of 5 µL via another stereotactic injection directly into the tumor site. We initially considered four different treatment groups: recombinant BMP-4 alone (*n* = 3), gPA alone (*n* = 3), gPA bound BMP-4 (*n* = 3), and a negative saline control (*n* = 4). After treatment, we measured tumor growth progression with bioluminescence imaging (BLI) every 2–3 days over the course of 1 month and found significant differences between experimental groups (Fig. 2B-C). While recombinant BMP-4 alone initially caused lower BLI levels, indicating a slow progression of tumor growth, after 29 days, the BLI levels of BMP-4 alone treated mice were similar to that of the saline control. We attribute this to BMP-4’s short half-life in vivo. BMP-4 presented on gPA, however, was especially effective, preventing an increase in BLI and maintaining significantly lower BLI levels over the course of the study. Interestingly, the gPA condition without BMP-4 also resulted in significantly lower BLI levels over time. We hypothesize that this is due to the free tri-sulfated monosaccharide group binding endogenous BMP-4 or other proteins that can also have an impact on tumor growth. In addition to reduced tumor growth, we observed a promising trend that mice treated with BMP-4 presented on gPA had higher survival rates when compared to the negative control over 100 days (Fig. 2D) – however, future studies will be needed to establish significance. We attribute the therapeutic efficacy of BMP-4 presented on gPA to the stabilizing effect of the PA nanostructure on BMP-4, which is able to protect the protein from degradation while also maintaining its bioactivity.

While BMP-4 presented on gPA was able to significantly reduce tumor growth and increase survival over 30 days and 100 days, respectively, we were interested if supplementing this treatment with a known chemotherapeutic that could further improve these outcomes, especially considering the high-risk of tumor recurrence developing from glioma precursor cells over time [56–59]. Because BMP-4 alone has the ability to differentiate stem-like glioma precursor cells into a non-stem-like phenotype [8, 13] that can be more susceptible to chemotherapy treatment [9], we hypothesized that a combinatorial treatment of PA-bound BMP-4 treatment and chemotherapy could be even more efficient at reducing tumor growth, especially at longer time points. To confirm this approach, we first assessed how pHGG cells in vitro would respond to recombinant BMP-4 in combination with chemotherapy, without any PA present. We found that both KNS42 cells and LCH-13 pGBM cells pre-incubated with 100 ng/mL of BMP-4 for 48 h and then treated with either vincristine (VCR) or vinblastine (VBL), both common clinical chemotherapeutic drugs previously used for malignant gliomas [60–62], had a significant decrease in cell viability compared to a no treatment control (Figure S3A-B). Interestingly, cell viability increased in all BMP-4 treated conditions when dorsomorphin, an established inhibitor of BMP-4 pathway activation [63–65], was used, suggesting that chemotherapeutic susceptibility is specifically triggered by BMP-4 activation of pSMAD1/5/8 pathway (Figure S3A-B).

**Fig. 3:**
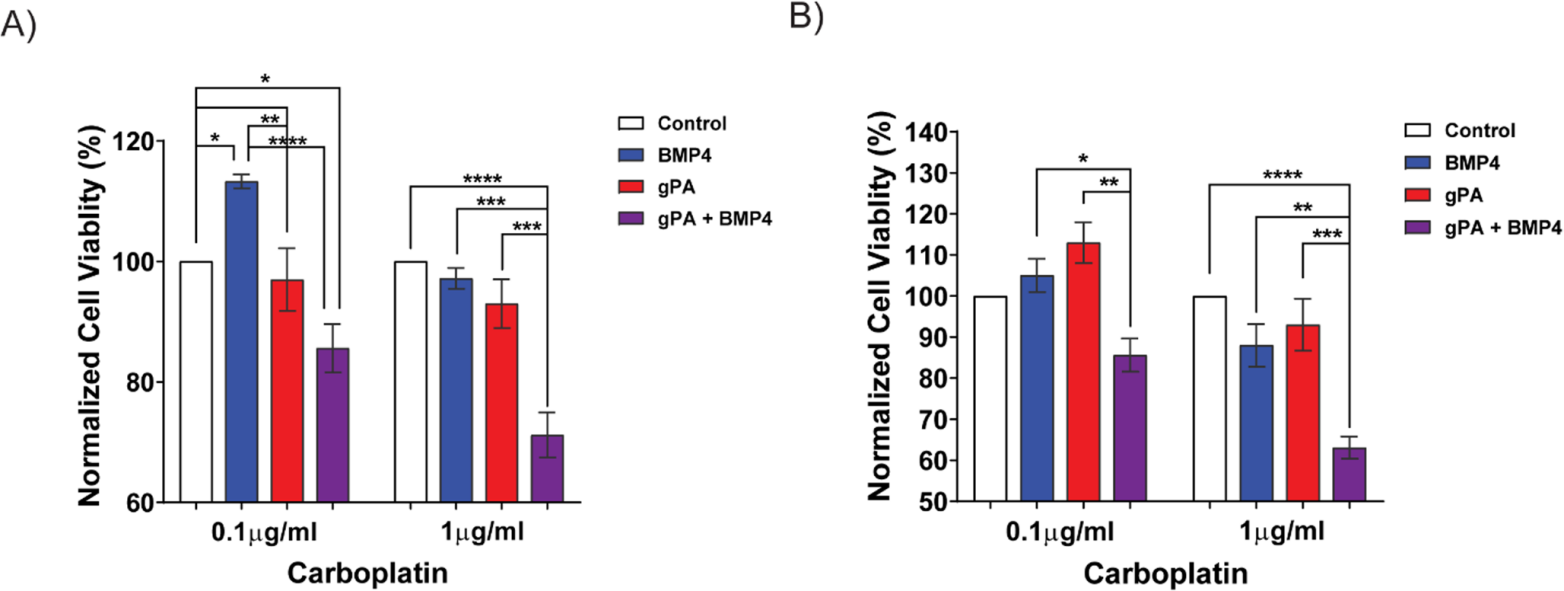
Combinatorial treatments of gPA bound BMP-4 and carboplatin reduce pediatric tumor cell viability. Normalized cell viability of (**A**) KNS42 cells and **(B)** CHLA200 cells treated with carboplatin and either BMP-4 (5 ng/mL), gPA (25 µM), or a combination of gPA (25 µM) and BMP-4 (5 ng/mL) in solution. For (**A-B**) a two-way ANOVA with multiple comparisions (α = 0.05) was performed: (*) P < 0.05, (**) P < 0.01, (***) P < 0.001, (****) P < 0.0001

After confirming that recombinant BMP-4 alone rendered pHGG cells more susceptible to chemotherapy, we next considered the impact that gPA bound BMP-4 had on KNS42 cell susceptibility to carboplatin treatment, another well-established chemotherapeutic drug used for malignant gliomas [66]. With carboplatin dosages derived from previous studies [66], we were encouraged to find a dose dependent impact of carboplatin on KNS42 cell viability after incubation with gPA bound BMP-4 (Fig. 3A). Similar results were demonstrated in CHLA200 cells (Fig. 3B). Because these in vitro studies indicated that gPA bound BMP-4 could increase pHGG chemosensitivity, we performed a preliminary study in which we delivered cisplatin [67] in combination with BMP-4 alone and gPA bound BMP-4 in an in vivo orthotopic xenograft pHGG SCID mouse model. We found that gPA bound BMP-4 in combination with cisplatin significantly reduced tumor growth over the course of 26 days when compared to a negative saline control as well as a combinatorial treatment of recombinant BMP-4 alone and cisplatin (Figure S4A). Additionally, we found that PA bound BMP-4 delivered with cisplatin had improved survival outcomes compared to the control (Figure S4B). However, despite significantly lower BLI levels indicating minimal tumor growth, the overall survival time was shorter than anticipated. We hypothesize that this was due to the chemotherapeutic cisplatin, a widely used platinum-based anticancer drug that is known to cause damage to the kidneys, liver, and nervous system, rather than tumor complications [68]. Future studies will optimize the combinatorial dosage and timing of cisplatin with gPA bound BMP-4. We anticipate that the coadministration of BMP-4 will likely reduce the total amount of cisplatin needed to maintain tumor suppression, reducing its cytotoxic effects while maintaining its therapeutic benefit. Additionally, we will consider delivering the chemotherapeutic systemically to more closely mimic the current clinical standard.

## Conclusions

We found that glycosylated peptide nanostructures (gPAs) can effectively present recombinant BMP-4 protein for pediatric high-grade glioma (pHGG) treatment. Because access to sufficient quantities of glycosylated peptide is essential for progress in translational glycobiology, we demonstrate here that the gPA molecule exhibits long-term storage stability and can be synthesized at scale to meet the demands of future in vivo studies. We believe that gPA binds to the heparan sulfate binding domain of BMP-4 to enhance its bioactivity, as we previously established with BMP-2 [24]. We established the importance of the sulfated glycan epitope in optimizing interactions with the heparan sulfate–binding domain. Our combined in vitro and in vivo results demonstrate that PA nanostructures are a promising BMP-4 delivery platform for clinical application in the treatment of brain tumors in pediatric patients. The local injection of our therapies via stereotactic injection could be replaced by convection enhanced delivery (CED) [69–71], one of the most effective therapeutic drug delivery methods for brain tumor treatment, and one that has already received FDA approval (SmartFlow Neuro Cannula, Regulation No. 21 CFR 882.4110). As such, we believe that our therapy can be administered via methods already widely used in the clinic for glioma patients of all ages. Future work will assess how BMP-4 presented on gPA can be combined with chemotherapeutics to enhance treatment of pHGG [72, 73], with the goal of reducing and ultimately eliminating the need for chemotherapy. Moreover, because gPA stabilizes BMP-4, it may enable lower dosages and less frequent administration of BMP-4, thereby improving quality of life for patients.

## Supplementary Information

Below is the link to the electronic supplementary material.

Supplementary Material 1

## Data Availability

The data that support the findings of this study are available from the corresponding author upon reasonable request.

## Etrovalhics App

All animal housing and procedures were performed in accordance with the Public Health Service Policy on Humane Care and Use of Laboratory Animals. All procedures were approved by the Northwestern University Institutional Animal Care and Use Committee.
