# Supplementary material for: Regenerative Glycopeptide Scaffolds Enhance BMP-4 Activity To Treat Pediatric Glioma
Source: Regen Eng Transl Med. 2025 Dec 19;11(4):1121–31. doi: 10.1007/s40883-025-00543-5 (PMC12852313; doi:10.1007/s40883-025-00543-5)
Supplement: Supplementary file 1 — Supplementary Material 1 [file 40883_2025_543_MOESM1_ESM.docx]

Supplemental Information

**Regenerative Glycopeptide Scaffolds Enhance BMP-4 Activity to Treat Pediatric Glioma**

Cara S. Smith^1,‡^, Timmy Fyrner^2,‡^, Nicholas A. Sather^2^, Mark T. McClendon^2^, Oscar A. Carballo-Molina^2^, Charles D. James^5^, Tadanori Tomita^4,5^, Guifa Xi^4,5,*^, Samuel I. Stupp^1,2,3,6,7,*^

^1^Department of Biomedical Engineering, Northwestern University, Evanston, IL 60208, USA.

^2^Center for Regenerative Nanomedicine, Northwestern University, Chicago, Illinois 60611, USA.

^3^Department of Chemistry, Northwestern University, Evanston, IL 60208, USA.

^4^Division of Pediatric Neurosurgery, Ann & Robert H. Lurie Children’s Hospital, Northwestern University Feinberg School of Medicine, Chicago, IL 60611, USA.

^5^Department of Neurological Surgery, Northwestern University Feinberg School of Medicine, Chicago, IL 60611, USA.

^6^Department of Materials Science and Engineering and Department of Chemistry Northwestern University, Evanston, IL 60208, USA.

^7^Department of Medicine, Northwestern University, Chicago, IL 60611, USA.

^‡^These authors contributed equally to this work.

**Methods**

**PA synthesis and purification.** **E_2_-PA** and **Alkyne-PA** were synthesized according to previously published protocols and consistent with data therein.^1^
The synthesis of **gPA** (Scheme S1) commenced from the commercially available 2-acetamido-1,3,4,6-tetra-*O*-acetyl-2-deoxy-β-D-glucopyranose (Biosynth, #MA06633). In a three-step manner *i.e.*, oxazoline formation, pyridinium p-toluene sulfonate (PPTS) promoted glycosylation with 2-chloroethanol, and azidation afforded the crystalline glycoside **2** in 47% over three steps (60 mmol scale). Subsequent Zemplén deacetylation, followed by final sulfation using Me_3_NSO_3_ generated the trisulfated monosaccharide **4** in 78% (8 mmol scale).^1^ The trisulfated glycoside was conjugated to the Alkyne-PA according to previously published protocol using a CuAAC reaction which after HPLC purification afforded the **gPA**^1^ in 72%.

**General synthetic procedure.** All reagent and chemicals were purchased form Millipore Sigma, if not otherwise stated. Dichloroethane was dried with pre-activated 4 Å molecular sieves. Thin layer chromatography (TLC) was carried out on Merck 60 F_254_ plates and developed with PAA staining solution [EtOH (95%, 740 mL), H_2_SO_4_ (conc., 28 mL), AcOH (100%, 8.4 mL), *p*-anisaldehyde (20 mL)]; AMC staining solution (ammonium molybdate (10 g), cerium(IV)sulfate (2 g), sulfuric acid (10%, 200 ml)); or PMA staining solution (phosphomolybdic acid (7 g), ethanol (100 ml)), followed by heating at ∼250 °C. Flash column chromatography (FC) was carried out on silica gel Merck 60 (40-63 μm).

**NMR Spectroscopy.** Proton nuclear magnetic resonance (^1^H) spectra were recorded on a Bruker Avance III HD 500MHz with a BBO Prodigy probe (sensitivity: ^1^H=1200, ^13^C=700) or a Bruker Neo 600 MHz system with a QCI-F cryoprobe (sensitivity: ^1^H=5000, ^13^C=800). Carbon nuclear magnetic resonance (^13^C) spectra were recorded on a Bruker Neo 600 MHz system with a QCI-F cryoprobe (sensitivity: ^1^H=5000, ^13^C=800), a Bruker Avance III HD 500MHz with a BBO Prodigy probe (sensitivity: ^1^H=1200, ^13^C=700) or a Bruker Avance III 500 MHz with a DCH CryoProbe (sensitivity: ^1^H=1800, ^13^C=1400). NMR spectra were recorded at 25 °C using CDCl_3_, CD_3_OD, D_2_O, or DMF-d_7_ as solvents. Chemical shifts are reported in part per million (ppm) with tetramethylsilane (TMS), the solvent residual peaks, or 5% v/v methanol (for D_2_O) as internal standards (CDCl_3_ (^1^H-NMR, δ = 7.26 ppm; ^13^C-NMR, δ = 77.2 ppm), D_2_O (^1^H-NMR, δ = 3. 34 ppm; ^13^C NMR, δ = 49.5 ppm), DMF-d7 (^1^H-NMR, δ = 8.03 ppm; ^13^C-NMR, δ = 163.2 ppm)).^2^ Structural assignment was performed using ^1^H-^1^H-gCOSY, ^1^H-^13^C-gHSCQAD, ^1^H-^1^H-TOCSY, and ^1^H-^13^C-gHMBCAD. Multiplicities are quoted as singlet (s), doublet (d), triplet (t), double of doublets (dd), double double of doublets (ddd), double of triplets (dt), apparent doublet (ad), apparent double of doublets (add), apparent double of triplets (adt), apparent triplet (at), quartet (q), and multiplet (m).

**Purity measurements.** LC-MS was performed on an Agilent 1200 system with an Agilent 6520 Q-TOF detector. All gradient methods followed: acetonitrile at 5% for 5 min at 50 µl min^–1^, 5–95% over 25 min at 50 µl min^−1^ followed by 95% for 5 min at 50 µl min^−1^. Ammonium hydroxide (0.1% v/v) was added to all solvents and a Phenomenex Gemini C-18, 100 x 1 mm; 5 µm was used.

**Accurate Mass Analysis.** High resolution mass spectra (HRMS) were recorded on an Agilent 6210A LC-TOF mass spectrometer in positive- or negative ion mode using electrospray ionization (ESI).

**Glycochemistry.**

**Scheme S1. Synthetic scheme to generate azido-functionalized trisulfated glycoside (4).** i) TMSOTf, DCE, ii) 2-chloroethanol, PPTS, DCE, Δ, iii) TBAB, NaN_3_, DMF, Δ, iv) K_2_CO_3_, MeOH, v) Me_3_NSO_3_, DMF, Δ.

**2-Azidoethyl 2-acetamido-3,4,6-tri-*O*-acetyl-2-deoxy-*β*-D-glucopyranoside (2)**

2-Acetamido-1,3,4,6-tetra-O-acetyl-2-deoxy-β-D-glucopyranose (50.00 g, 128.4 mmol) **(1)**, *R_f_* = 0.62 (EtOAc/MeOH 9:1), was purchased from Biosynth® and dissolved in dry dichloroethane (500 mL). Trimethylsilyl trifluoromethanesulfonate (TMSOTf) (25.57 mL, 141.26 mmol) was added under inert atmosphere at room temperature. After 1.5 hours, the reaction mixture quenched with triethylamine (20 mL, 270.2 mmol) and concentrated. FC (EtOAc) gave the intermediate 2-Methyl-(3,4,6-tri-O-acetyl-1,2-dideoxy-α-D-glucopyrano)-[2,1-D]-2-oxazoline^3^ as a colorless syrup. *R_f_* = 0.58 (EtOAc/MeOH 9:1); ^13^C NMR (125 MHz, CDCl_3_): δ 170.6, 170.0, 169.2, 166.6, 99.4 (C-1), 70.3 (C-3), 68.5 (C-4), 67.5 (C-5), 65.0 (C-2), 63.4 (C-6), 20.9 (-CH_3_), 20.9 (-CH_3_), 20.8 (-CH_3_), 14.0 (oxazoline -CH_3_); ^1^H NMR (500 MHz ,CDCl_3_): δ 5.83 (d, 1H, *J =* 7.3 Hz, H-1), 5.11 (at, 1H, *J =* 2.4 Hz, *J =* 2.4 Hz, H-3), 4.78 (ddd, 1H, *J =* 9.2, 2.1, 1.3 Hz, H-4), 4.19 – 3.69 (m, 3H, H-2, H-6_ab_), 3.46 (dt, 1H, *J =* 8.9, 4.3 Hz, H-5), 1.97 (s, 3H, -CH_3_), 1.95 (s, 3H, -CH_3_), 1.94 (s, 3H, oxazoline -CH_3_), 1.94 (s, 3H, -CH_3_). The intermediate oxazoline, *R_f_* = 0.37 (EtOAc), was dissolved in dry dichloroethane (500 mL) whereupon 2-chloroethanol (86.15 mL, 1.28 mol) and PPTS (3.23 g, 12.8 mmol) were added at room temperature and heated to 70°C. After 6.5 hours the reaction mixture was cooled down to room temperature and the reaction was quenched with pyridine (50 mL, 620 mmol) followed by evaporation and co-concentration with toluene. The crude was dissolved in dichloromethane (250 mL) and washed with 1M HCl (aq.), NaHCO_3_ (sat.aq.), once with H_2_O, dried over MgSO_4_ (s), filtered and evaporated to generate the intermediate 2-chloroethyl 2-acetamido-3,4,6-tri-*O*-acetyl-2-dideoxy-β-D-glucopyranoside^4^ as a colorless syrup. Crystallization from EtOAc/n-heptane provided the compound as colorless needles. *R_f_* = 0.48 (EtOAc); ^13^C NMR (125 MHz, CDCl_3_): δ 170.8, 170.7, 170.5, 169.4, 101.1 (C-1), 72.1 (C-3), 72.0 (C-5), 69.7 (-CH_2_), 68.6 (C-4), 62.0 (C-6), 54.7 (C-2), 43.0 (-CH_2_), 23.4 (-CH_3_), 20.8 (-CH_3_), 20.7 (-CH_3_), 20.6 (-CH_3_); ^1^H NMR (500 MHz ,CDCl_3_): δ 5.58 (d, 1H, *J =* 8.7 Hz, -NH), 5.32 (dd, 1H, *J =* 10.7, 9.3 Hz, H-3), 5.07 (dd, 1H, *J =* 10.0, 9.3 Hz, H-4), 4.79 (d, 1H, *J =* 8.4 Hz, H-1), 4.26 (dd, 1H, *J =* 12.3, 4.8 Hz, H-6a), 4.16 – 4.12 (m, 1H, H-6b), 4.12 – 4.07 (m, 1H, -CH_2_), 3.87 (adt, 1H, *J =* 10.7, 8.4 Hz, H-2), 3.78 (ddd, 1H, *J =* 11.3, 6.8, 5.9 Hz, -CH_2_), 3.72 (ddd, 1H, *J =* 10.0, 4.8, 2.5 Hz, H-5), 3.64 (dd, 2H, *J =* 6.2, 4.9 Hz, -CH_2_), 2.09 (s, 3H, -CH_3_), 2.04 (s, 3H, -CH_3_), 2.03 (s, 3H, -CH_3_), 1.97 (s, 3H, -CH_3_). ESI-MS: [M + H]^+^ calcd for C_16_H_25_ClNO_9_, 410.1218; found 410.1211. The crude 2-chlorethyl glycoside was dissolved in DMF (100 mL), sodium azide (13.42 g, 206.4 mmol), and TBAB (6.65 g, 20.64 mmol) was added and the reaction was stirred over night at 70°C. The reaction was monitored with LC-MS since the 2-chloroethyl glycoside and the 2-azidoethyl glycoside has identical *R_f_*-values. The reaction mixture was cooled down to room temperature and the diluted with EtOAc (500 mL). The organic phase was washed multiple time with brine, H_2_O, dried with MgSO_4_ (s), filtered, and evaporated. Crystallization (EtOAc/n-heptane) generated the titled product **(2)**^5^ as colorless needles (25.27 g, 60.69 mmol, 47% over three steps). *R_f_* = 0.48 (EtOAc). ^13^C NMR (125 MHz, CDCl_3_): δ 170.8, 170.7, 170.5, 169.4, 100.5 (C-1), 72.1 (C-3), 72.0 (C-5), 68.7 (C-4), 68.4 (-CH_2_), 62.0 (C-6), 54.9 (C-2), 50.6 (-CH_2_), 23.4 (-CH_3_), 20.8 (-CH_3_), 20.7 (-CH_3_), 20.6 (-CH_3_);^1^H NMR (500 MHz ,CDCl_3_): δ 5.71 (d, 1H, *J =* 8.6 Hz, -NH), 5.37 (dd, 1H, *J* = 10.7, 9.3 Hz, H-3), 5.08 (dd, 1H, *J* = 10.0, 9.3 Hz, H-4), 4.84 (d, 1H, J = 8.4 Hz, H-1), 4.26 (dd, 1H, J = 12.3, 4.8 Hz, H-6b), 4.16 (dd, 1H, *J* = 12.2, 2.5 Hz, H-6a), 4.05 (ddd, 1H, *J* = 10.9, 4.7, 3.3 Hz, -CH_2_), 3.82 (dt, 1H, *J* = 10.6, 8.5 Hz, H-2), 3.78 – 3.65 (m, 2H, H-5, -CH_2_), 3.51 (ddd, 1H, *J* = 13.4, 8.5, 3.3 Hz, -CH_2_), 3.27 (ddd, 1H, *J* = 13.4, 4.7, 3.1 Hz, -CH_2_), 2.09 (s, 3H, -CH_3_), 2.04 (s, 3H, -CH_3_), 2.03 (s, 3H, -CH_3_), 1.96 (s, 3H, -CH_3_). ESI-MS: [M + H]^+^ calcd for C_16_H_25_N_4_O_9_, 417.1622; found 417.1615.

**2-Azidoethyl 2-acetamido-2-deoxy-*β*-D-glucopyranoside (3)**

The titled compound was prepared by dissolving 2-Azidoethyl 2-acetamido-3,4,6-tri-O-acetyl-2-dideoxy- β-D-glucopyranoside **(2)**^5^ (15.50 g, 37.26 mmol) in MeOH (50 mL) followed by the addition of NaOMe (3.12 g, 55.84 mmol). After 1 hour, the reaction was neutralized with Dowex-H^+^ ion exchange resin, filtered and concentrated to generate 2-Azidoethyl 2-acetamido-2-dideoxy- β-D-glucopyranoside **(3)**^5^ (14.91 g, 51.37 mmol, 92%) as a white powder. ^13^C NMR (125 MHz, CDCl_3_): δ 169.7, 102.5 (C-1), 78.1 (C-4), 76.2 (C-3), 72.1 (C-5), 69.3 (C-6), 62.8 (-CH_2_O), 57.2 (C-2), 51.8 (-CH_2_N), 23.1 (-CH_3_); ^1^H NMR (500 MHz, CD_3_OD) δ 4.48 (d, 1H, *J* = 8.4 Hz, H-1), 4.03 (ddd, 1H, *J* = 11.0, 5.4, 3.3 Hz, H6a), 3.89 (dd, 1H, *J* = 11.9, 2.2 Hz, -CH_2_O), 3.71 – 3.65 (m, 3H, H-2, H6b, -CH_2_O), 3.53 – 3.39 (m, 2H, H-3, -CH_2_N), 3.37 – 3.23 (m, 3H, H-4, H-5, -CH_2_N), 1.98 (s, 3H). ESI-MS: [M + H]^+^ calcd for C_10_H_19_N_4_O_6_, 291.1305; found 219.1307.

**2-Azidoethyl 2-acetamido-3,4,6-tri-*O*-sulfo-2-deoxy-*β*-D-glucopyranoside (4)**

The titled compound was synthesized according to previously published protocols by dissolving 2-Azidoethyl 2-acetamido-2-dideoxy- β-D-glucopyranoside **(3)** (3.15 g, 10.85 mmol) in dry DMF (25 mL) whereupon Me_3_NSO_3_ (7.55 g, 54.25 mmol) was added and the reaction mixture heated to 80°C. After 24 hours, the reaction cooled down to room temperature and quenched with MeOH (30 mL) followed by evaporation and co-concentration with toluene. FC (ACN/H_2_O/NH_3_ (20%) 6:1:0.5) gave the trisulfated monosaccharide **(4)**^1^ as a colorless solid (4.49 g, 8.46 mmol, 78%). ^1^H NMR (500 MHz, D_2_O) δ 4.77 (d, *J* = 8.4 Hz, 1H, H-1), 4.62 (dd, *J* = 11.5, 2.3 Hz, 1H, H-6a), 4.53 (dd, *J* = 10.2, 8.8 Hz, 1H, H-4), 4.27 (dd, *J* = 9.8, 8.8 Hz, 1H, H-3), 4.16 (dd, *J* = 11.5, 8.1 Hz, 1H, H-6b), 4.10 (ddd, *J* = 11.4, 5.5, 2.9 Hz, 1H, OCH_2_CH_2_N), 4.00 – 3.95 (m, 2H, H-2, H-5), 3.85 (ddd, *J* = 11.4, 7.8, 2.9 Hz, 1H, OCH_2_CH_2_N), 3.58 – 3.49 (m, 1H, OCH_2_CH_2_N), 3.43 (ddd, *J* = 13.6, 5.5, 2.9 Hz, 1H, OCH_2_CH_2_N), 2.02 (s, 3H, CH_3_). ^13^C NMR (125 MHz, D_2_O) δ 175.4 (CO), 101.0 (C-1), 78.9 (C-4), 75.1 (C-3), 73.2 (C-2), 69.8 (OCH_2_CH_2_N), 68.3 (C-6), 55.1 (C-5), 50.9 (OCH_2_CH_2_N), 23.1 (CH_3_). ESI-MS: [M - H]^-^ calcd for C_10_H_17_N_4_O_15_S_3_, 528.9852; found 528.9870. *For purity see Figure S21.*

**Trisulfated glycopeptide PA (gPA)**

Alkyne-terminated PA^1^ (2.00 g, 1.66 mmol) and sulfated azido-functionalized compound **(4)** (966 mg, 1.82 mmol) were dissolved in DMF (10 mL) followed by the addition of 1M CuSO_4_ (497 μL, 497 μmol) and sodium ascorbate (2.95 g, 14.91 mmol). The reaction mixture was shaken overnight whereupon H_2_O (10mL) was added. The pH was monitored with pH-paper and adjusted to ~8 using ammonia solution (25%). The reaction mixture was treated 5 times with Chelex® 100, where 5 grams were added and the mixture was shaken for 5 minutes, filtered and washed with H_2_O (5 mL). The solution was evaporated to dryness to generate the crude gPA. The crude gPA was dissolved in 0.1 % NH_4_OH (aqueous ammonia) solution, then filtered through a 0.2 µm syringe filter. High-performance liquid chromatography (HPLC) purification was conducted using a Waters Prep 150 LC system, with a gradient of water/acetonitrile containing 0.1% NH_4_OH (aq.), utilizing a Phenomenex Gemini C18 column. Target compound was identified using direct injection electrospray ionization mass spectroscopy on an Agilent 6520 Q-TOF system. The volatile solvents were evaporated, and the remaining material was lyophilized, resulting in the target sulfated glycopeptide PA (gPA)^1^ as a white powder (2.23 g, 1.28 mmol, 72%). *NMR was consistent with previously published data^1^, see Figure S26-S29.* ESI-MS: [M - 2H]^2-^ calcd for C_69_H_120_N_14_O_31_S_3_, 867.3625; found 867.3641; *m/z where z = 2*. *For purity see Figure S26 and LCMS analysis S31, S32. See NMR Figure S30 and LCMS in Figure S32 for stability study.*

**PA sample preparation*.*** Lyophilized PA powder was dissolved at a concentration of 1 wt% in 150 mM NaCl, 3 mM KCl solution. The PA solution was pH adjusted to approximately 7.4-7.6, horn sonicated, and then annealed for 30 minutes at a temperature of 80 °C and slow cooled at a rate of 1 °C/minute back to room temperature. PA mixtures were formed by mixing the desired molar concentrations of PA prior to annealing. For *in vivo* studies, lyophilized PA powder was similarly prepared, but initially dissolved at a concentration of 1 wt% in 0.9% sterile saline solution. Samples were also bath sonicated to maintain sterility.

**Rheology.** All rheology experiments were measured on an Anton-Paar MCR 302 rheometer using cone-plate geometry. Viscosity flow curves were collected using a CP50-1 fixture. PA samples were gently poured onto the bottom plate to minimize shear before measurement, and careful consideration was taken to remove all bubbles within the material. The viscosity values are reported at 10 s^-1^ shear rate. Gel rheology was measured with a CP25-2 fixture with a final salt concentration of 2.5 mM CaCl_2_ and 150 mM NaCl to mimic cerebrospinal fluid ion concentration. Following a 10-minute equilibration period, the gel modulus was measured at 0.1% strain and 10 rad/s.

**Cell culture.** Pediatric CHLA200 glioblastoma cells were obtained from the Children's Oncology Group Cell Culture and Xenograft Repository. Pediatric KNS42 cells were purchased from the JCRB (Japanese Cancer Research Resources) cell bank. LCH-13-pGBM cells were primary cultured cells under IRB protocol 2005-12252 (Principal investigator: Dr. Tadanori Tomita). All cells were propagated as monolayers in Dulbecco’s modified Eagle’s medium (DMEM, Cat#11965-092) supplemented with 10% fetal bovine serum (FBS, Gibco, Cat#10082147) at 37 °C with 5% CO_2_.

***In vitro* studies.** KNS42 cells in the presence of serum were treated with either BMP-4, gPA, or gPA + BMP-4. BMP-4 stock solution was prepared at 10 μg/mL. gPA stock solution was prepared at 2.5 μM. For BMP-4 only treatments, 2 μL of BMP-4 stock solution was mixed with 98 μL of cell culture media. For treatments containing both BMP-4 and gPA, 2 μL of BMP-4 stock solution was mixed with 98 μL of gPA stock solution. 20 μL of each prepared treatment was added to 2 mL of complete culture media, resulting in a final BMP-4 concentration of 2 ng/mL across condition. Cells for differentiation studies were stained with glial fibrillary acidic protein (GFAP) and DAPI. Cells for proliferation studies were stained with Ki67. Both the total number of cells and the number of Ki67+ cells were quantified from representative images at 24, 48, and 72 hours after treatment. Images were captured on a Leica DMR-HC upright microscope (Leica Microsystem Inc., Buffalo Groove, IL, USA) and analyzed using OpenLab 5.0 software (PerkinElmer, Waltham, MA, USA).

**Western blot.** pHGG cells were treated with one of the following treatments: BMP-4 (5 ng/ml), gPA (25 μM) or gPA (25 μM) + BMP-4 (5 ng/ml). At the desired timepoint, equal amounts of cell lysate were resolved by sodium dodecyl sulfate–polyacrylamide gel electrophoresis and transferred to nitrocellulose membranes (Bio-Rad, Hercules, CA, USA). Blocking was performed for 60 min with 5% nonfat dry milk in Tris-buffered saline and Tween 20, followed by blotting with primary antibodies overnight at 4 °C. After washing with Tris-buffered saline and Tween 20, membranes were incubated for 1 h at room temperature with horseradish peroxidase (HRP) conjugated donkey anti-rabbit antibody (sc-2305, 1:5000), and signal was detected with enhanced chemiluminescence substrate (Bio-Rad Laboratories).

**Enzyme-linked immunosorbent assay (ELISA).** PA samples were prepared as previously described. After annealing and gradual cooling, 100 µL of E2 PA or gPA were mixed with 200 µL of DMEM medium (Gibco, 11320) supplemented with 5 ng/mL of human recombinant BMP-4 protein (ThermoFisher, AF-120-05ET). As a negative control, 300 µL of medium alone (no treatment) was included. As a positive control, 300 µL of medium containing 5 ng/mL BMP-4 was used. To mimic physiological conditions, the samples was maintained in an incubator at 37 °C and 5% CO₂. Samples were collected at the indicated time points and stored at −20 °C. BMP-4 levels were quantified using a Human BMP-4 ELISA Kit (Abcam, ab231930) following the manufacturer’s instructions. We used GraphPad Prism (version 10.2.3) for plotting and statistical evaluation. Each point was compared separately by an Ordinary one-way ANOVA and a Multiple Comparisons Test.

**Epitope presentation calculations.** Epitope presentation on a peptide amphiphile (PA) nanofiber surface can be as high as approximately 1 × 10¹⁴ epitopes per cm² [9]. The glycosylated PA (gPA) described in this study can present a comparable density of ~1 × 10¹⁴ epitopes per cm², equivalent to approximately one monosaccharide per nm². Based on a read-across from the structurally related bone morphogenetic protein 2 (BMP-2), which has a predicted molecular mass of ~13 kDa (monomer) and dimensions of 7 × 3.5 × 3 nm,[10] the largest face corresponds to an area of ~25 nm². If this surface is involved in the interaction with gPA, approximately 25 monosaccharide units would be required to noncovalently bind one BMP-4 homodimer on the nanofiber surface.

In the orthotopic intracranial xenograft tumor model, 1.67 mM gPA was loaded with 1.28 pM BMP-4. Given that 1.67 mM gPA contains sufficient monosaccharide residues to bind up to 66.8 μM BMP-4, more than 99% of the available binding sites on the gPA remain unoccupied. Because gPA is non-anticoagulant, these unoccupied sites do not present a safety concern but rather offer the potential to further increase BMP-4 loading if required.

**IACUC standard statement.** All animal housing and procedures were performed in accordance with the Public Health Service Policy on Human Care and Use of Laboratory Animals. All procedures were approved by the Northwestern University Institutional Animal Care and Use Committee.

**PA in vivo intracranial distribution study.** All surgical instruments were cleaned and sterilized by pressurized steam sterilization in an autoclave prior to each surgical procedure, and a hot bead sterilizer was used to maintain instrument sterility throughout procedures. On the day of surgeries, 6-8-week-old NOD scid mice from Taconic were anesthetized intraperitoneally with a xylazine/ketamine mixture. Buprenorphine SR was administered for pain maintenance. Lubricating ophthalmic ointment was applied to the eyes, and then the surgical site was shaved and scrubbed with Betadine followed by two separate ethanol pads. Recirculating warm water blankets and deltaphase isothermal pads were used to maintain heat support through operation and recovery. After surgery preparation, mice were mounted in a stereotactic frame. A midline scalp incision was used to expose the skull, and then a 1 mm burr hole was made approximately 1.0 mm cranial from the coronal suture. A 26G needle attached to a 25 μL syringe was then inserted 1 mm back from bregma, 2 mm to the right, and 3 mm into the cortex and left to rest for 5 minutes before PA injection. Mice were injected at a final volume of 10 μL via stereotactic injection. Treatments were injected at a rate of 0.23 μL/minute. PAs were co-assembled with 1 mol% of Cy3-labelled E_2_-PA for visualization. Free Cy3 dye administered at the same concentration as present in the E_2_-PA fibers was used as a control. At the desired timepoint, animals were sacrificed, and the brains were extracted and frozen in optimal cutting temperature (OCT) compound. A Leica cryostat was used to section the tissue in the coronal plane in 40 μm thick slices. Images were captured on a Leica DMR-HC upright microscope (Leica Microsystem Inc., Buffalo Groove, IL, USA).

***In Vivo* orthotopic xenograft intracranial tumor model.** All surgical instruments were cleaned and sterilized by pressurized steam sterilization in an autoclave prior to each surgical procedure, and a hot bead sterilizer was used to maintain instrument sterility throughout procedures. On the day of each procedure, NU/NU nude mice from Charles River were anesthetized intraperitoneally with a xylazine/ketamine mixture. Buprenorphine SR was administered for pain maintenance. Lubricating ophthalmic ointment was applied to the eyes, and then the surgical site was shaved and then scrubbed with Betadine followed by two separate ethanol pads. Recirculating warm water blankets and deltaphase isothermal pads were used to maintain heat support through the operation and recovery. For tumor inoculation, NU/NU Nude mice were mounted in a stereotactic frame. A midline scalp incision was used to expose the skull, and then a 1 mm burr hole was made approximately 1.0 mm cranial from the coronal suture. A 26G needle attached to a 25 μL syringe was then inserted 1 mm back from bregma, 2 mm to the right, and 3 mm into the cortex and left to rest for 5 minutes before tumor inoculation. 5 μL of 500,000 KNS42 cells stably expressing the luciferase gene were then administered via stereotactic injection over a period of 10 minutes. After injection, the needle was left to rest for 5 minutes. The needle was then withdrawn at a rate of 1.0 mm/minute. The wound was then sutured with 4-0 nylon. Ear-tagging was used to identify mice.

7 days after tumor inoculation, mice where then treated with either saline, BMP-4, gPA, or gPA + BMP-4. BMP-4 stock solution was prepared at a concentration of 0.01 μg/μL in 4 mM HCl with 0.1% BSA in 1x PBS. PA stock solution was prepared at a concentration of 5 mM following previously described methods. To prepare treatments, gPA stock solution was mixed with 0.9% saline solution in a 1:2 ratio, resulting in a final concentration of 1.67 mM. A BMP4 stock solution of 100 μg/mL was mixed with 0.9% saline solution in a 1:2 ratio, resulting in a final concentration of 33.3 ng/μL. gPA + BMP-4 was prepared by mixing stock gPA solution, stock BMP-4 solution, and 0.9% saline solution in a 1:1:1 ratio. Treatments were administered via stereotactic injection in a final volume of 5 μL. Prior to PA injection, animals were anesthetized intraperitoneally with a xylazine/ketamine mixture. Buprenorphine SR was also administered for pain maintenance. Lubricating ophthalmic ointment was applied to the eyes, and then the surgical site was shaved and then scrubbed with Betadine followed by two separate ethanol pads. Recirculating warm water blankets and deltaphase isothermal pads were used to maintain heat support through the operation and recovery. The previous incision was re-opened, and the previous burr hole made in the skull was used to access the tumor. A 22G needle attached to a 25 μL syringe was inserted into the tumor using the stereotactic frame. The needle was positioned at the same coordinates as tumor inoculation. After 5 minutes of rest, 5 μL of treatment was injected. After infusion, the needle was left for an additional 5 minutes before withdrawing. The wound was closed with 4-0 nylon sutures.

Mice were euthanized with CO_2_ asphyxiation followed by cervical dislocation when they became moribund (e.g., >20% weight loss, neurologic symptoms, or evidence of pain/distress). Brains were harvested and fixed with 10% paraformaldehyde in PBS overnight and switched to PBS prior to embedding. The tissue was sectioned onto slides and stained with H&E. Tissue preparation was conducted at the Mouse Histology and Phenotyping Laboratory (MHPL), Northwestern University Feinberg School of Medicine.

**Bioluminescence Imaging (BLI)**. Prior to imaging, animals were anesthetized with 1-2% isoflurane and injected intraperitoneally with 150 mg/kg D-luciferin. 10 minutes after D-luciferin administration, animals were transferred into an IVIS® imaging system. Signals were followed until a peak emission was obtained. After 15 minutes, animals were returned to their cages and monitored until recovery from anesthesia.

***In vitro* chemotherapeutic studies.** pHGG cells were cultured *in vitro* following previously described procedures. BMP-4 (Gibco) was added in solution for variable amounts of time, followed by the addition of 1 μg/mL vincristine (VCR) or 2 μg/mL vinblastine (VBL). An inhibitor of pSMAD1/5/8, dorsomorphin, was also selectively added at a concentration of 2 μM. Protein was extracted following treatment and analyzed using Western blot. For experiments with Carboplatin, KNS42 or CHLA200 cells were treated with different concentrations of Carboplatin in combination with either BMP-4 (5 ng/mL), gPA (25 μM), or gPA (25 μM) + BMP-4 (5 ng/mL).

***In vivo* orthotopic xenograft intracranial tumor model with chemotherapeutic.** All surgical instruments were cleaned and sterilized by pressurized steam sterilization in an autoclave prior to each surgical procedure, and a hot bead sterilizer was used to maintain instrument sterility throughout procedures. On the day of each procedure, NOD scid were anesthetized intraperitoneally with a xylazine/ketamine mixture. Buprenorphine SR was administered for pain maintenance. Lubricating ophthalmic ointment was applied to the eyes, and then the surgical site was shaved and then scrubbed with Betadine followed by two separate ethanol pads. Recirculating warm water blankets and deltaphase isothermal pads were used to maintain heat support through the operation and recovery.

For tumor inoculation, NOD scid mice were mounted in a stereotactic frame. A midline scalp incision was used to expose the skull, and then a 1 mm burr hole was made approximately 1.0 mm cranial from the coronal suture. A 26G needle attached to a 25 μL syringe was then inserted 1 mm back from bregma, 2 mm to the right, and 3 mm into the cortex and left to rest for 5 minutes before tumor inoculation. 5 μL of KNS42 cells were then administered via stereotactic injection over a period of 10 minutes. After injection, the needle was left to rest for 5 minutes. The needle was then withdrawn at a rate of 1.0 mm/minute. The wound was then sutured with 4-0 nylon. Ear-tagging was used to identify mice.

7 days after tumor inoculation, mice were then treated with either saline, BMP-4, gPA, or gPA + BMP-4 in combination with Cisplatin. gPA and BMP-4 stock solutions were prepared as previously described. gPA stock solution was mixed with 0.9% saline solution in a 1:2 ratio, resulting in a final concentration of 1.67 mM. BMP-4 was mixed with 0.9% saline solution in a 1:2 ratio, resulting in a final concentration of 3.33 ng/μL. Cisplatin stock solution was prepared at a concentration of 1 μg/μL in 1x PBS. BMP-4 + Cisplatin treatment was prepared by mixing BMP-4 stock solution, Cisplatin stock solution, and 0.9% saline solution in a 1:1:1 ratio. gPA + BMP4 + Cisplatin treatment was prepared by mixing gPA stock solution, BMP-4 stock solution, and Cisplatin stock solution in a 1:1:1 ratio. The final dosing of BMP-4 was 33.3 ng. The final dosing of Cisplatin was 3.33 μg. Cisplatin dosing was determined from previous literature.^6,7^ Prior to PA injection, animals were anesthetized intraperitoneally with a xylazine/ketamine mixture. Buprenorphine SR was also administered for pain maintenance. Lubricating ophthalmic ointment was applied to the eyes, and then the surgical site was shaved and then scrubbed with Betadine followed by two separate ethanol pads. Recirculating warm water blankets and deltaphase isothermal pads were used to maintain heat support through the operation and recovery. The previous incision was re-opened, and the previous burr hole made in the skull was used to access the tumor. A 22G needle attached to a 25 μL syringe was inserted into the tumor using the stereotactic frame. The needle was positioned at the same coordinates as tumor inoculation. After 5 minutes of rest, 10 μL of treatment was injected. After infusion, the needle was left for an additional 5 minutes before withdrawing. The wound was closed with 4-0 nylon sutures.

**Statistical Analysis.** Data analysis was performed with GraphPad Prism software (version 9.5.0). Comparisons among three or more groups were conducted with one-way or two-way ANOVA with a Tukey’s multiple comparisons test, unless otherwise indicated. For BLI measurements, data from each treatment group was collected and used to plot normalized bioluminescence values at each imaging time-point. Two-way ANOVA with Tukey’s multiple comparisons test was used for statistical analysis. For survival analysis, we used the Kaplan-Meier estimator to analyze survival curves and to determine median survival values. Statistical differences in survival among groups were determined using the log-rank (Mantel-Cox) test. The statistical tests and parameters used for each experiment are reported in the corresponding figure legends. All error bars shown in graphs represent the standard error mean unless otherwise indicated.

**References**

1. S.S. Lee, T. Fyrner, F. Chen, Z. Álvarez, E. Sleep, D.S. Chun, J.A. Weiner, R.W. Cook, R.D. Freshman, M.S. Schallmo, Sulfated glycopeptide nanostructures for multipotent protein activation, Nature nanotechnology 12(8) (2017) 821.
2. Gottlieb, Hugo E., Kotlyar, V., and Nudelman, Abraham, NMR Chemical Shifts of Common Laboratory Solvents as Trace Impurities, J. Org. Chem. 62(21): 7512-7515. (1997).
3. Wittmann, V., Lennartz, D, Copper(II)-Mediated Activation of Sugar Oxazolines: Mild and Efficient Synthesis of β-Glycosides of N-Acetylglucosamine, European Journal of Organic Chemistry. 8: 1363-1367. (2002).
4. Sukhova, E.V., Dubrovskii, A.V., Tsvetkov, Yu. E., Nifantiev, N.E., Synthesis of oligosaccharides related to the HNK-1 antigen. 5.* Synthesis of a sulfo-mimetic of the HNK-1 antigenic trisaccharide**, Russian Chemical Bulletin. 56(8): 1655-1670. (2007).
5. Eklind, K., Gustafsson, R., Tidén, A.K., Norberg, T., Aberg, P.M., Large-Scale Synthesis of a Lewis b Tetrasaccharide Derivative, its Acrylamide Copolymer, and Related DI- and Trisaccharides for Use in Adhesion Inhibition Studies with *Helicobacter Pylori*, Journal of Carbohydrate Chemistry. 15(9): 1161-1178. (1996).
6. Zisman, N., Dos Santos, N., Johnstone, S., Tsang, A., Bermudes, D., Mayer, L., Tardi, P, Optimizing Liposomal Cisplatin Efficacy through Membrane Composition Maninpulations, Chemotherapy Research and Practice. (2011).
7. Shikanov, A., Shikanov, S., Vaisman, B., Golenser, J., Domb, A., Cisplatin Tumor Biodistribution and Efficacy after Intratumoral Injection of a Biodegradable Extended Release Implant, Chemotherapy Research and Practice. (2011).
8. C. Zhang, E. A. Nance, P. Mastorakos, J. Chisholm, S. Berry, C. Eberhart, B. Tyler, H. Brem, J.S. Suk, J. Hanes, Convection enhanced delivery of cisplatin-loaded brain penetrating nanoparticles cures malignant glioma in rats. J Control Release. 263(10) (2017) 112-119.
9. Silva GA, Czeisler C, Niece KL, Beniash E, Harrington DA, Kessler JA, Stupp SI. Selective differentiation of neural progenitor cells by high-epitope density nanofibers. Science. 2004 Feb 27;303(5662):1352-5. doi: 10.1126/science.1093783. Epub 2004 Jan 22. PMID: 14739465.
10. Scheufler C, Brinker A, Bourenkov G, Pegoraro S, Moroder L, Bartunik H, Hartl FU, Moarefi I. Structure of TPR domain-peptide complexes: critical elements in the assembly of the Hsp70-Hsp90 multichaperone machine. Cell. 2000 Apr 14;101(2):199-210.

**Supplemental Figures**


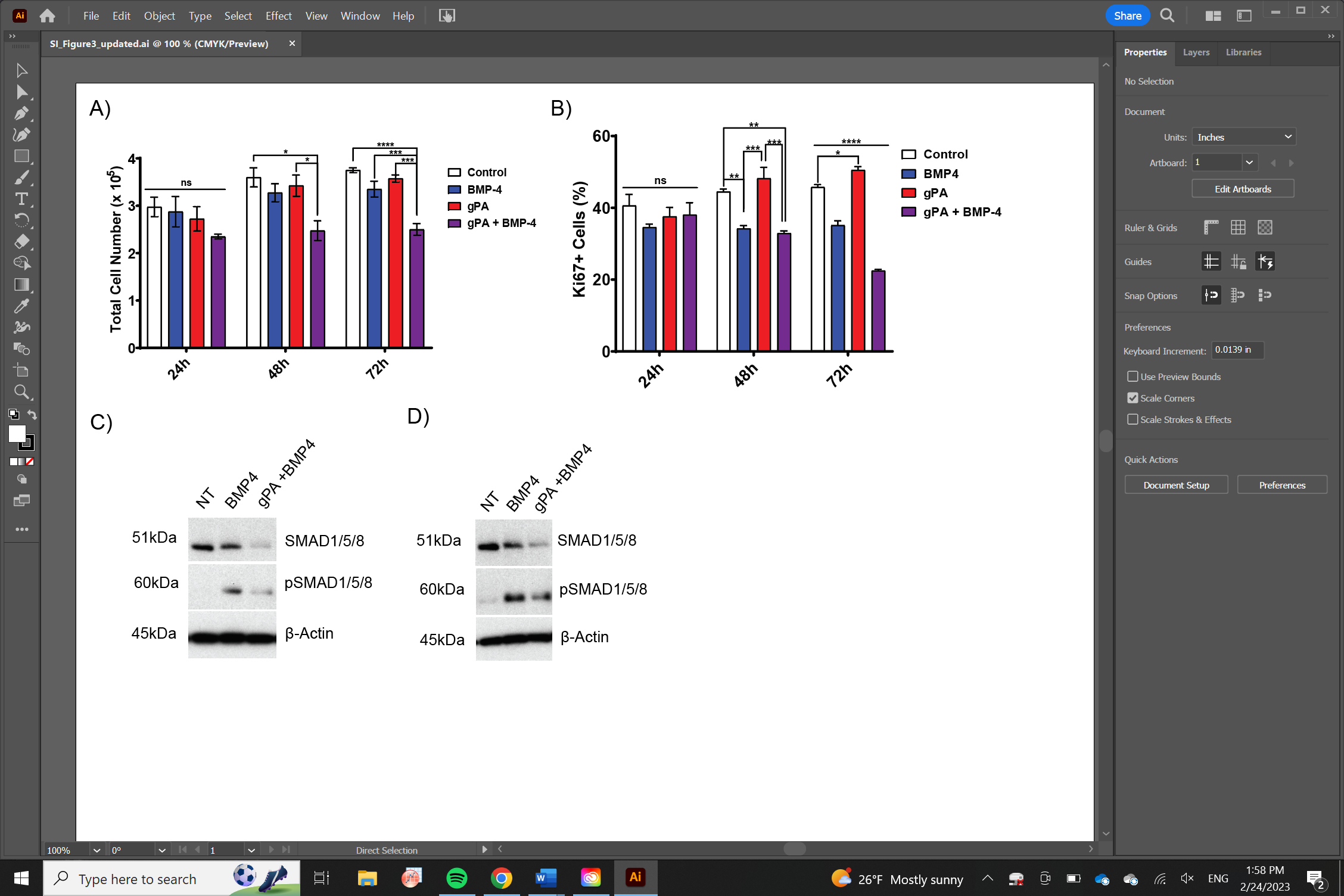


**Figure S1. gPA bound BMP-4 reduces cell number and proliferation and activates BMP-4 receptor pathway. (A)** Total cell number of and **(B)** percent Ki67 positive KNS42 cells treated with BMP-4, gPA, or a combination of BMP-4 and gPA for 24, 48, and 72 hours. Representative Western blot of SMAD1/5/8, pSMAD1/5/8, and β-actin of CHLA200 cells treated with either free BMP-4 (5 ng/mL) or a combination of gPA (25 μM) and BMP-4 (5 ng/mL) for **(C)** 1 hour and **(D)** 6 hours**.** For **(A-B)** one-way ANOVA with Tukey’s multiple comparison’s test was performed: (*) P < 0.05, (**) P < 0.01, (***) P < 0.001, (****) P < 0.0001.

**
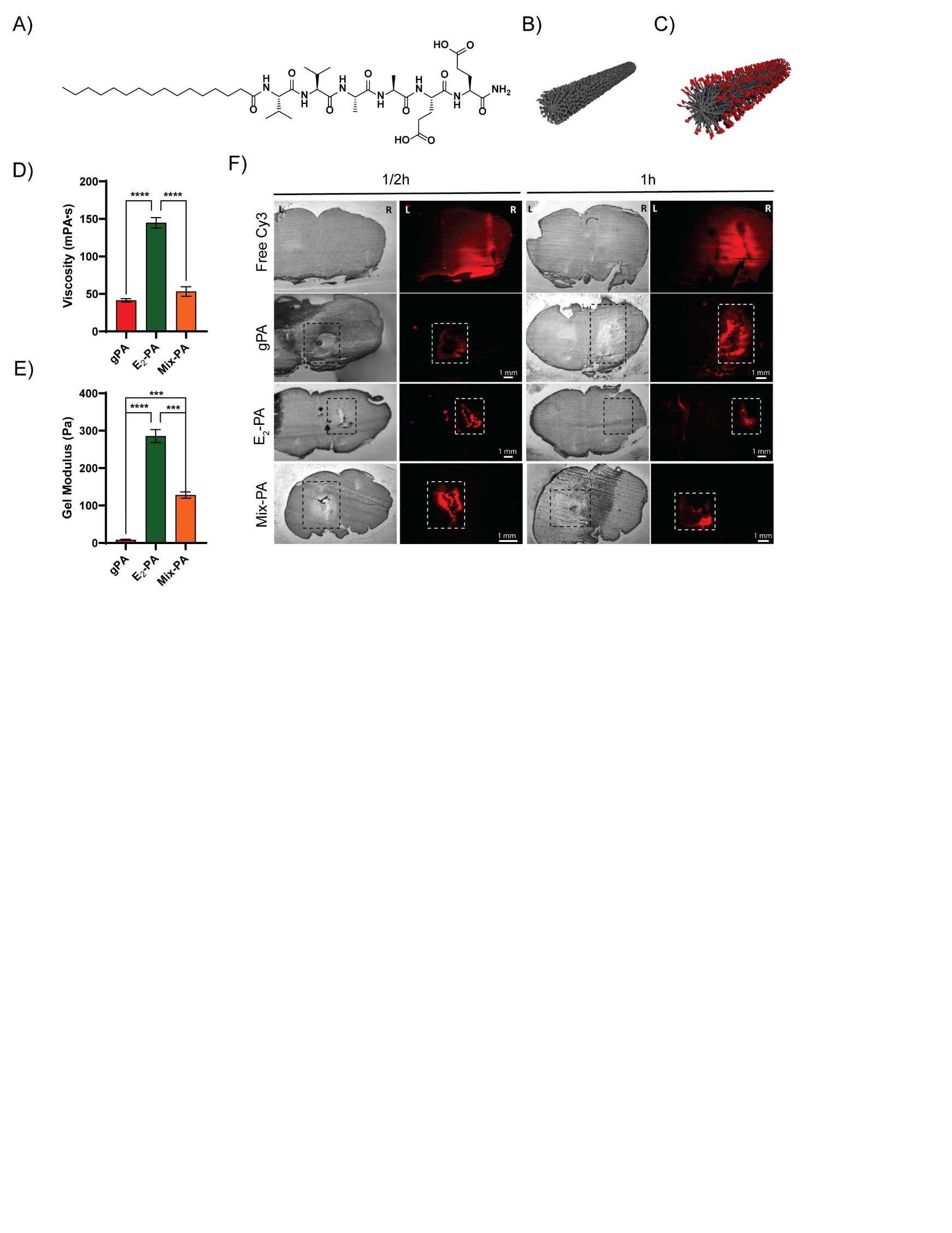
**

**Figure S2. Intracranial gPA distribution and rheological properties. (A)** E_2_-PA molecular structure. **(B)** E_2_-PA nanofiber rendering. **(C)** Mix-PA, comprised of a 50:50 molar ratio of gPA:E_2_-PA, nanofiber rendering. Tri-sulfated monosaccharide groups are represented in red. The **(D)** viscosity and **(E)** gel modulus of PA solutions gelled with a cerebrospinal fluid mimetic solution comprised of 2.5 mM CaCl_2_ and 150 mM NaCl. **(F)** Representative brightfield and fluorescent micrographs of healthy mouse brain tissue stereotactically injected with either free Cy3 or Cy3-labelled PA nanofibers after a ½ hour or 1 hour. For **(D-E)** a one-way ANOVA with a Tukey’s mulitple comparisions test (α = 0.05) was performed: (*) P < 0.05, (**) P < 0.01, (***) P < 0.001, (****) P < 0.0001.


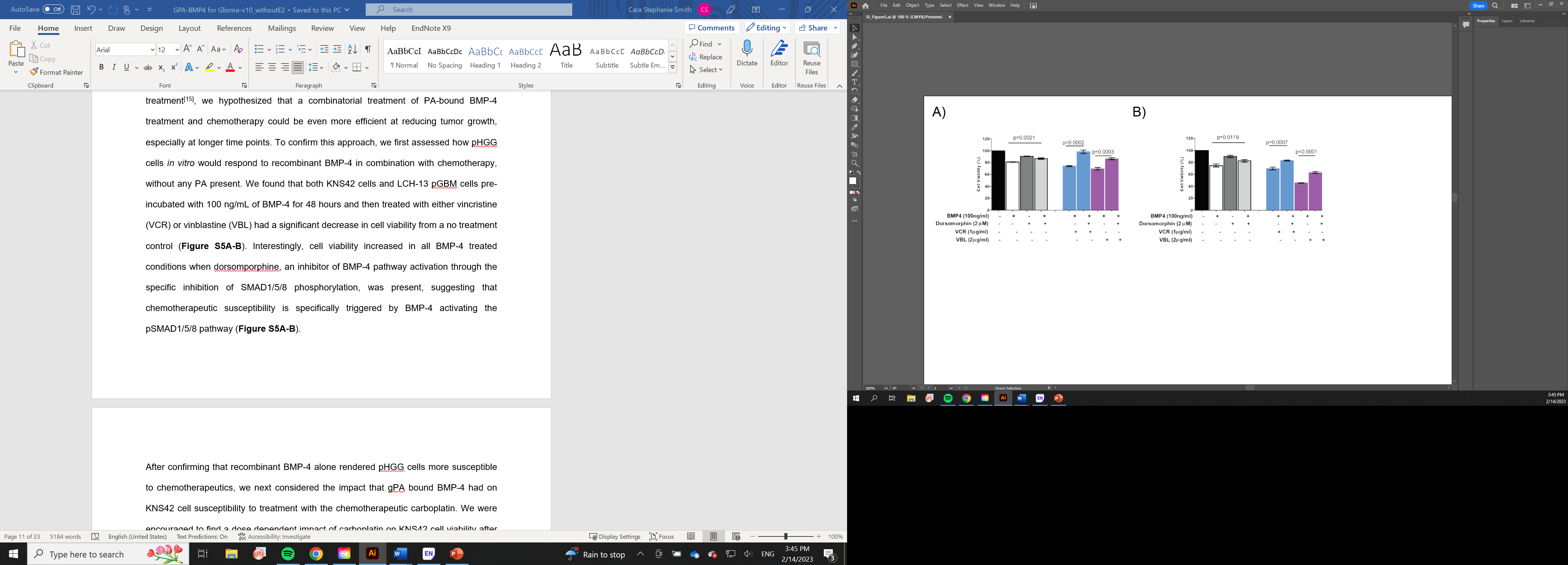


**Figure S3. BMP-4 in combination with chemotherapeutics reduces pHGG cell viability. (A)** KNS42 and **(B)** LCH-13 pGBM cells treated with either BMP-4, BMP-4 and vincristine (VCR), or BMP-4 and vinblastine (VBL) either with or without the addition of the SMAD1/5/8 phosphorylation inhibitor, dorsomorphin.


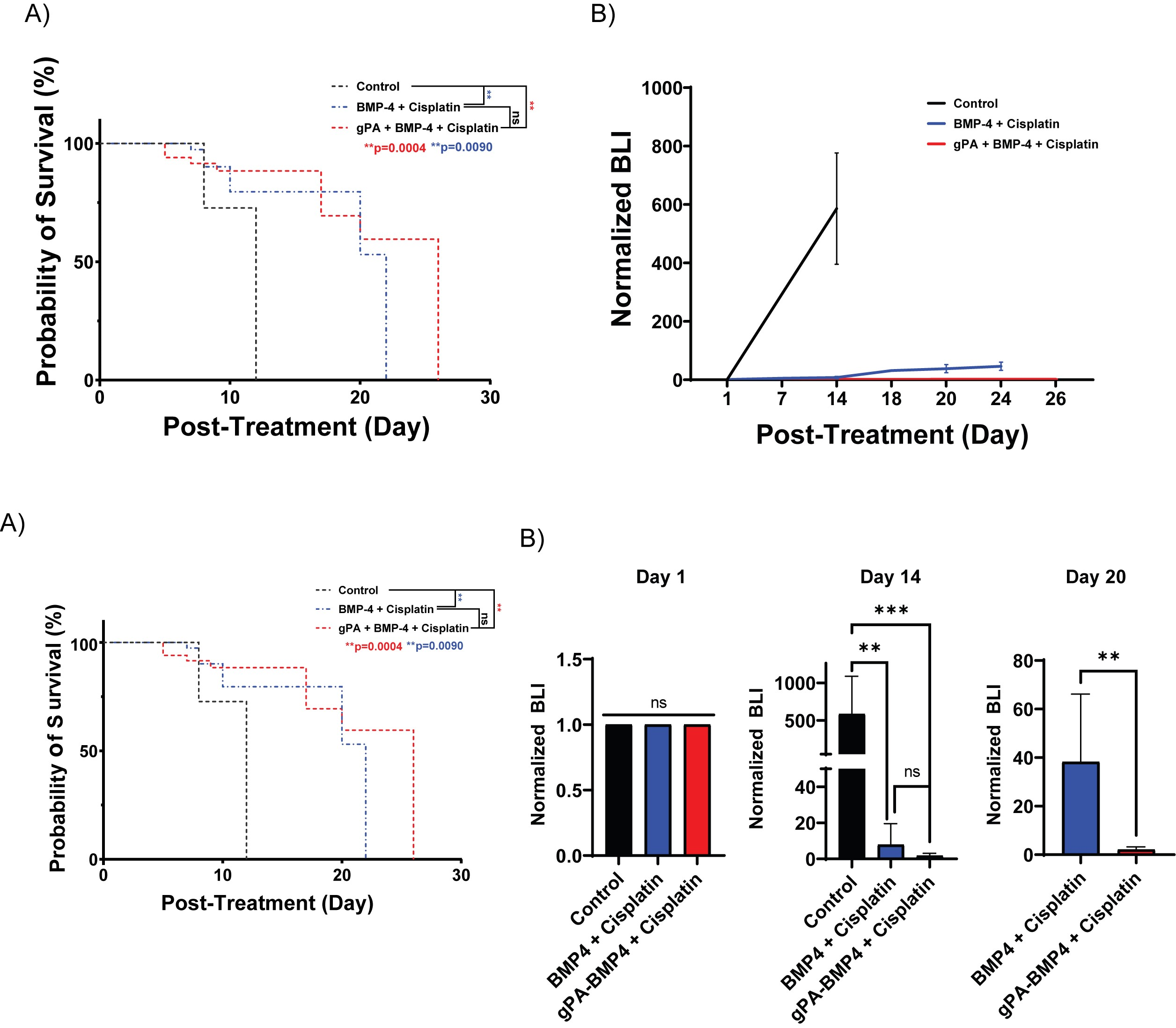


**Figure S4. gPA, BMP-4, and cisplatin treatments in an orthotopic xenograft intracranial pediatric high grade glioma (pHGG) tumor model. (A)** Probability of survival and **(B)** Normalized BLI over 26 days. For **(A)** a log-rank (Mantel-Cox) test was performed. For **(B)** one-way ANOVA with a Tukey’s multiple comparisons test was used for comparisons between 3 groups and an unpaired t test was used for comparison between 2 groups.

**
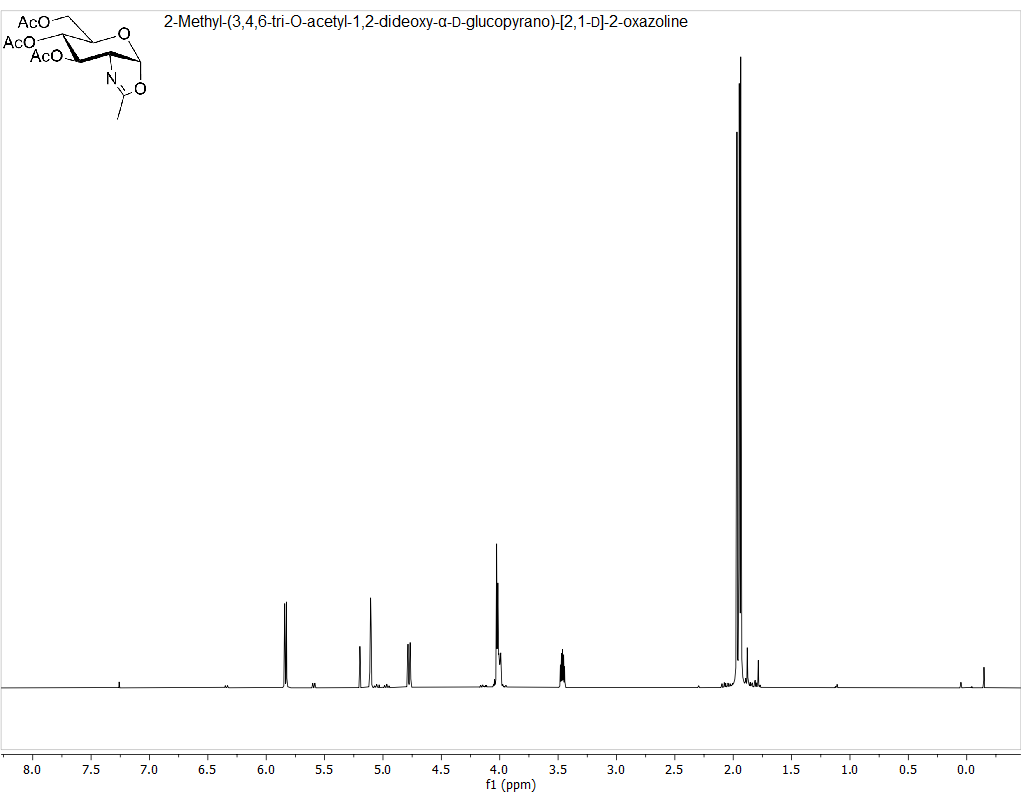
**

**Figure S5. Proton-NMR in CDCl_3_ of oxazoline-intermediate.**

**
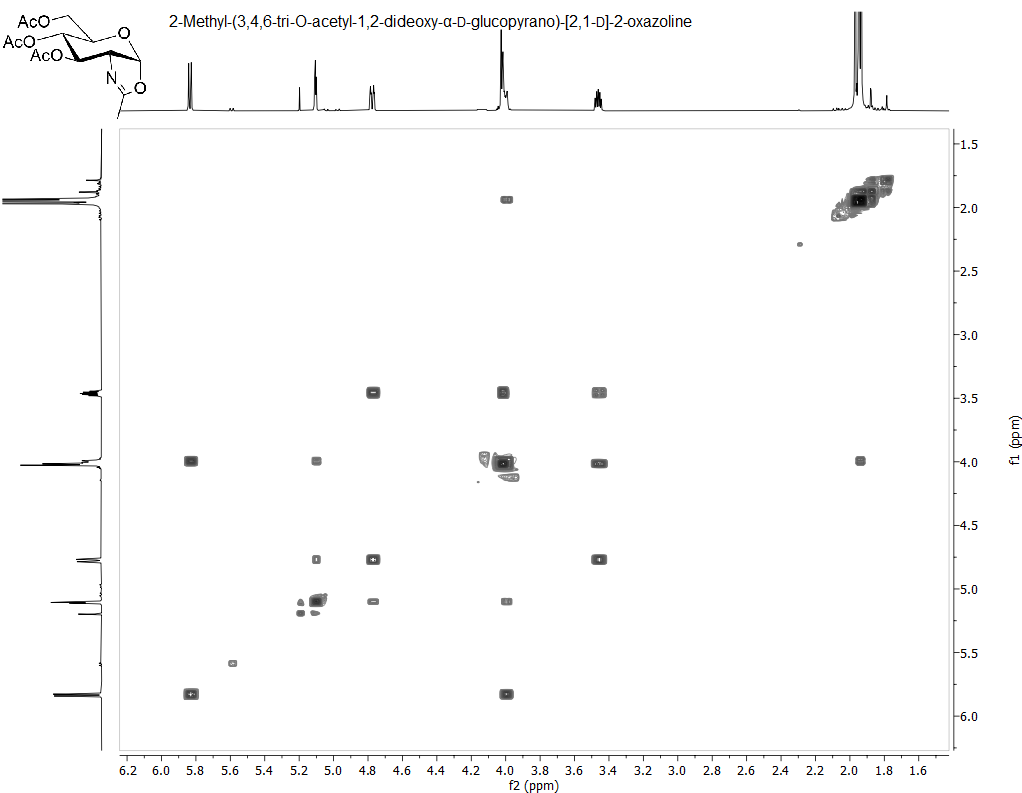
**

**Figure S6. ^1^H-^1^H-COSY NMR in CDCl_3_ of oxazoline-intermediate.**

**
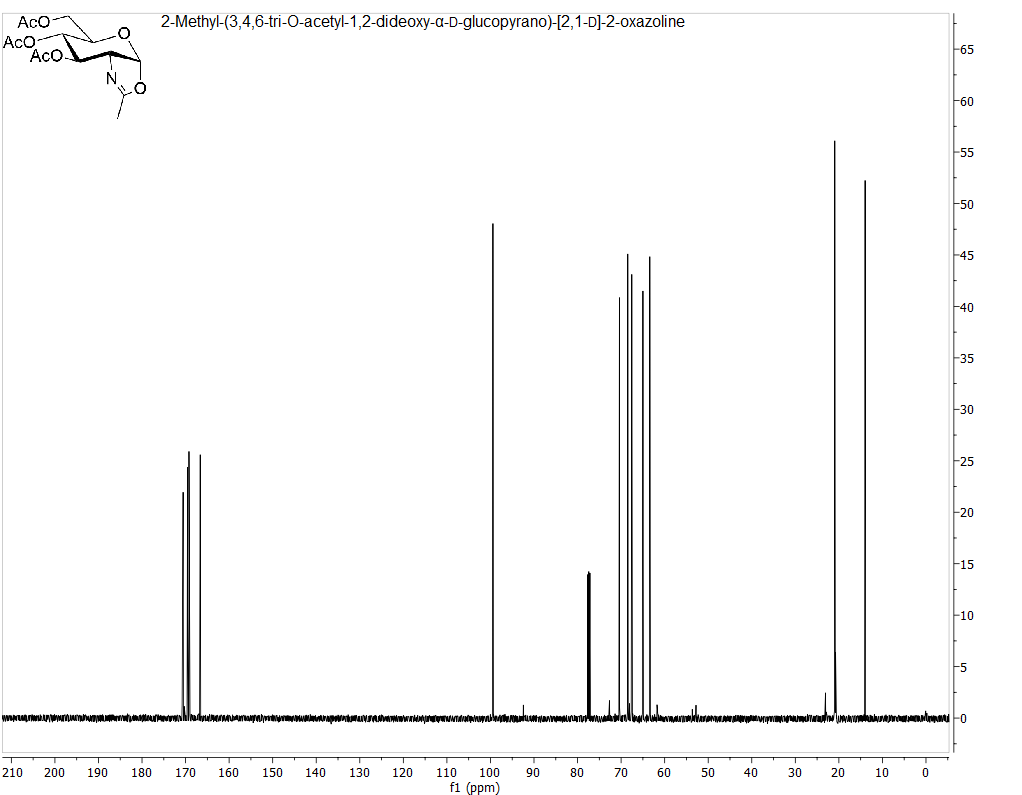
**

**Figure S7. Carbon-NMR in CDCl_3_ of oxazoline-intermediate.**

**
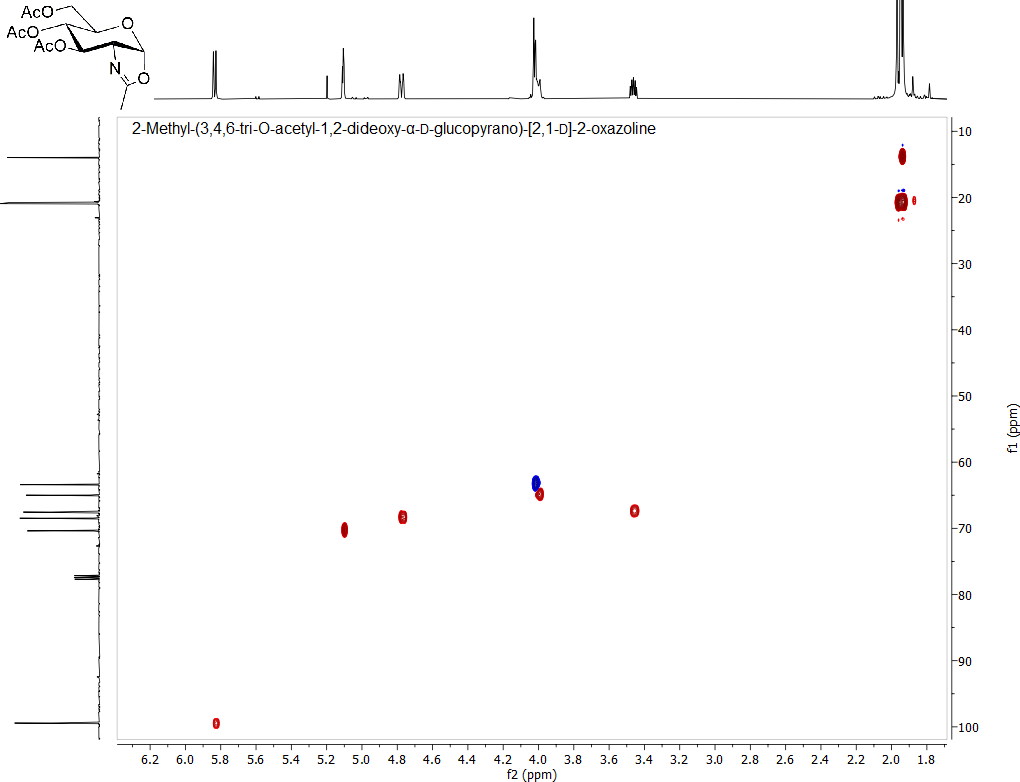
**

**Figure S8. ^1^H-^13^C-HSQC NMR in CDCl_3_ of oxazoline-intermediate.**

**
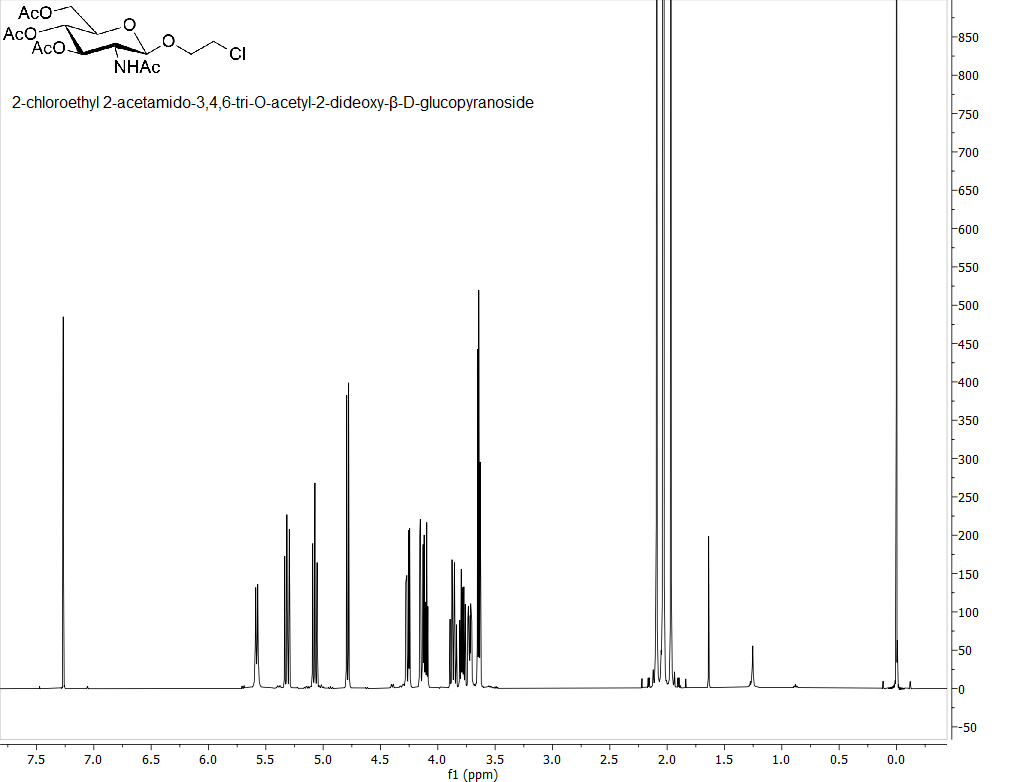
**

**Figure S9. Proton-NMR in CDCl_3_ of intermediate 2-chloroethyl 2-acetamido-3,4,6-tri-*O*-acetyl-2-dideoxy-*β*-D-glucopyranoside.**

**
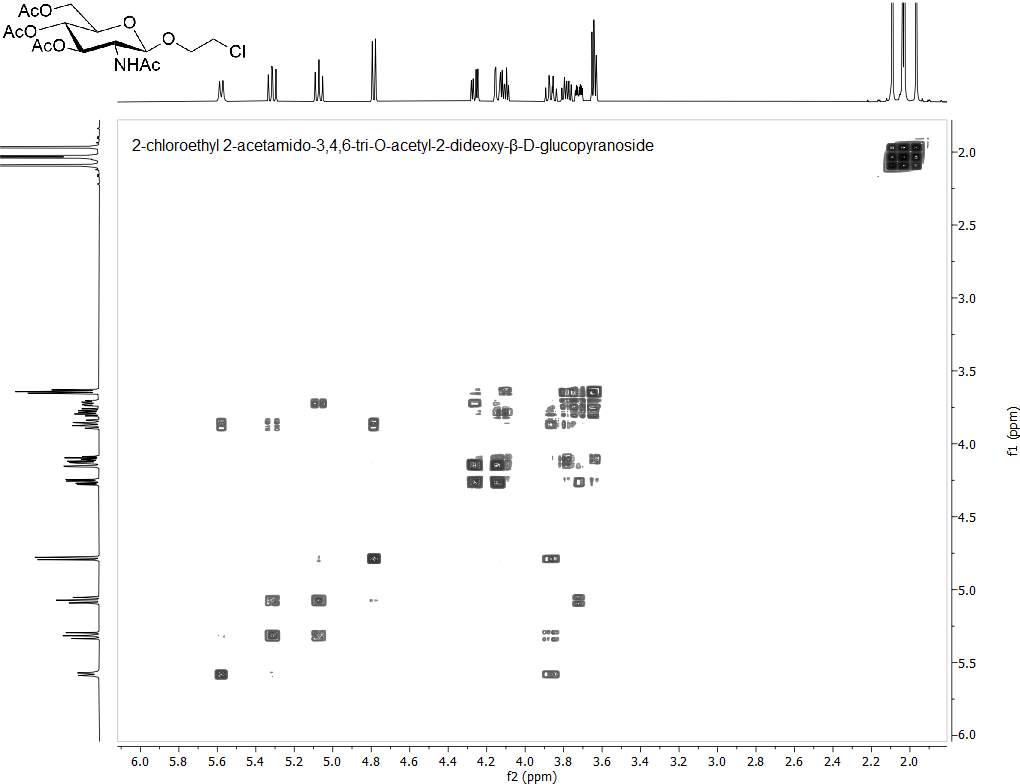
**

**Figure S10. ^1^H-^1^H-COSY NMR in CDCl_3_ of intermediate 2-chloroethyl 2-acetamido-3,4,6-tri-*O*-acetyl-2-dideoxy-*β*-D-glucopyranoside.**

**
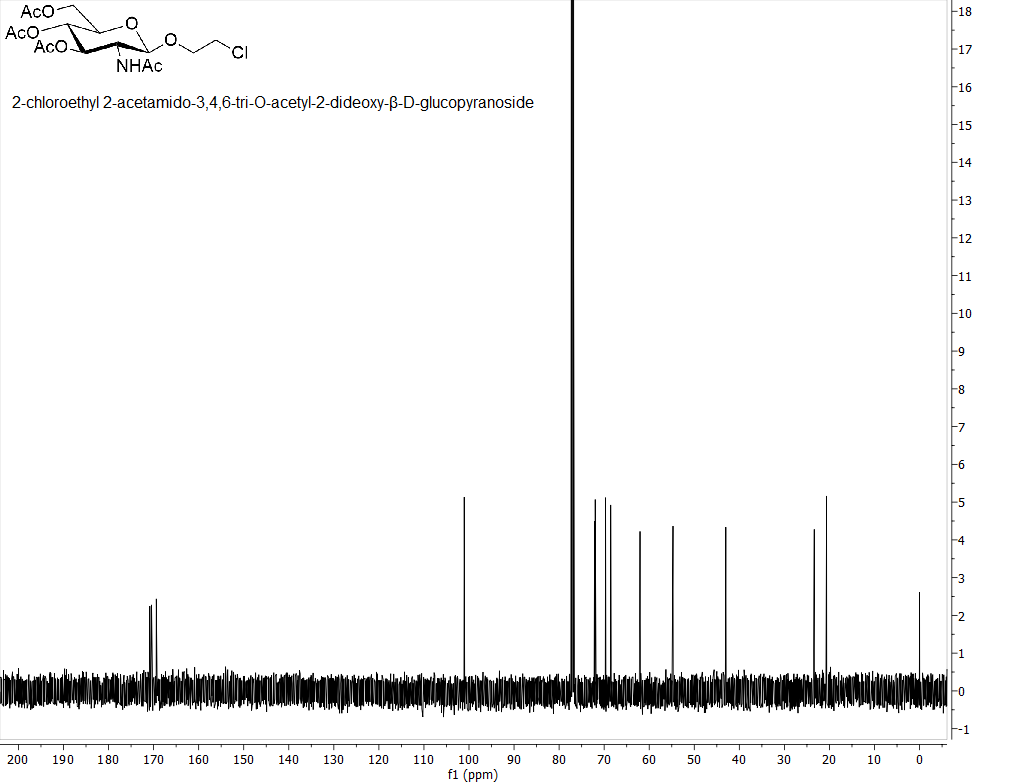
**

**Figure S11. Carbon-NMR in CDCl_3_ of intermediate 2-chloroethyl 2-acetamido-3,4,6-tri-*O*-acetyl-2-dideoxy-*β*-D-glucopyranoside.**

**
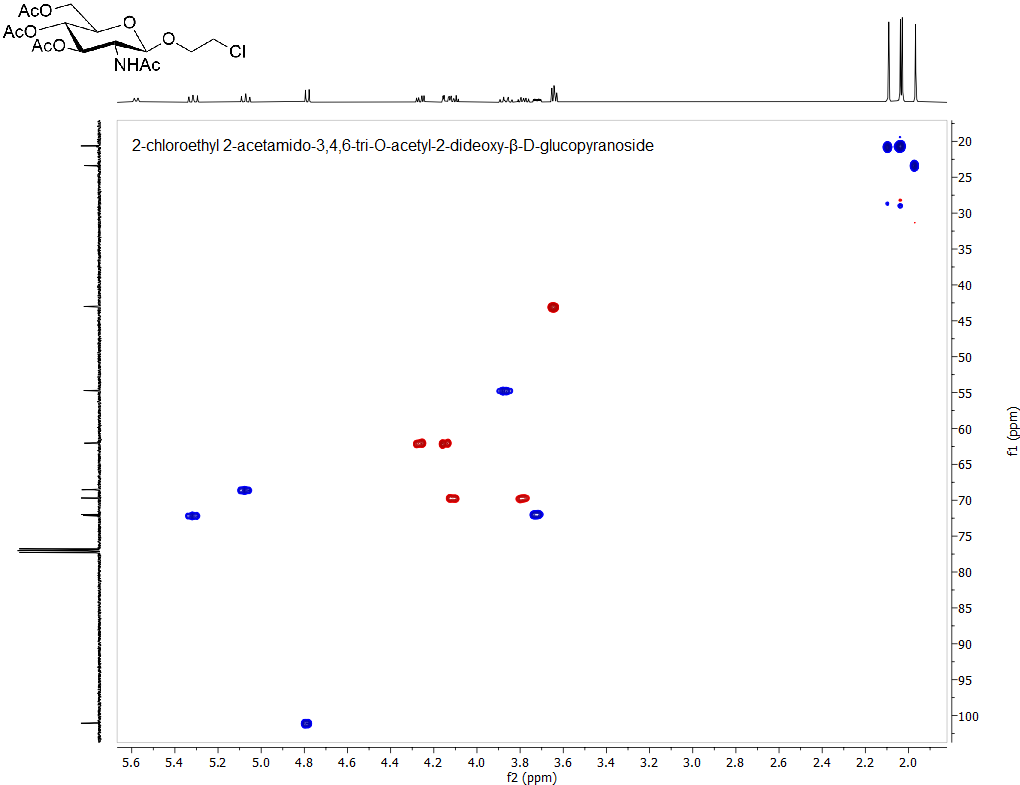
**

**Figure S12. ^1^H-^13^C-HSQC NMR in CDCl_3_ of intermediate 2-chloroethyl 2-acetamido-3,4,6-tri-*O*-acetyl-2-dideoxy-*β*-D-glucopyranoside.**

**
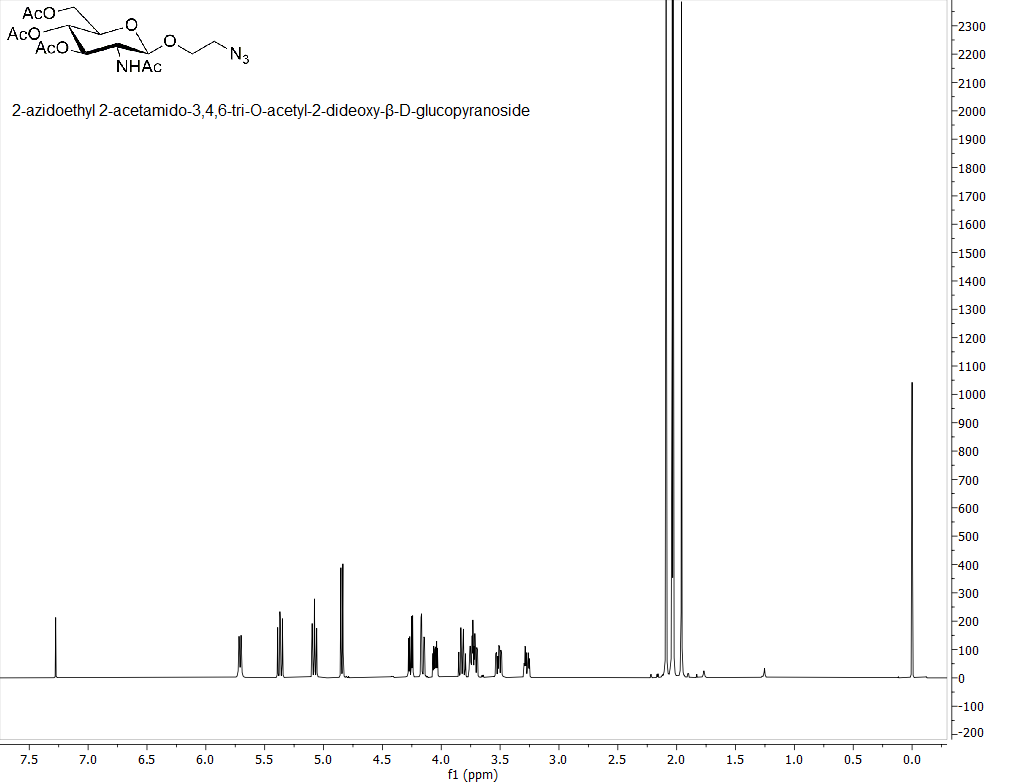
**

**Figure S13. Proton NMR in CDCl_3_ of 2-azidoethyl 2-acetamido-3,4,6-tri-*O*-acetyl-2-dideoxy-*β*-D-glucopyranoside (2).**


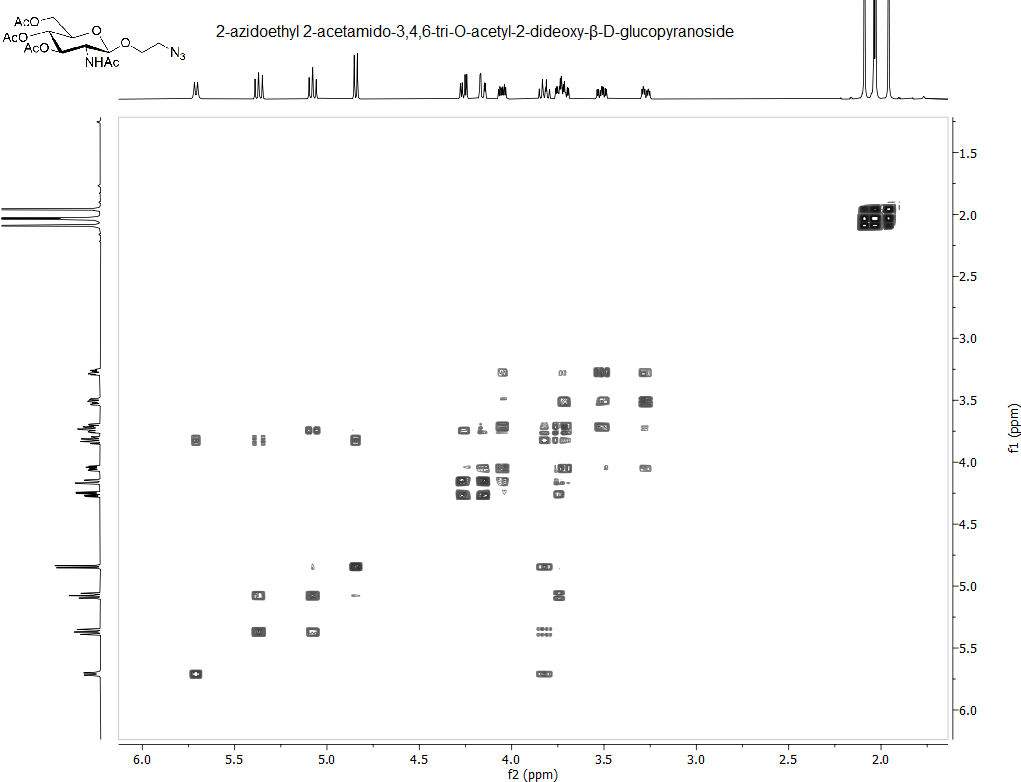


**Figure S14. ^1^H-^1^H-COSY NMR in CDCl_3_ of 2-azidoethyl 2-acetamido-3,4,6-tri-*O*-acetyl-2-dideoxy-*β*-D-glucopyranoside (2).**

**
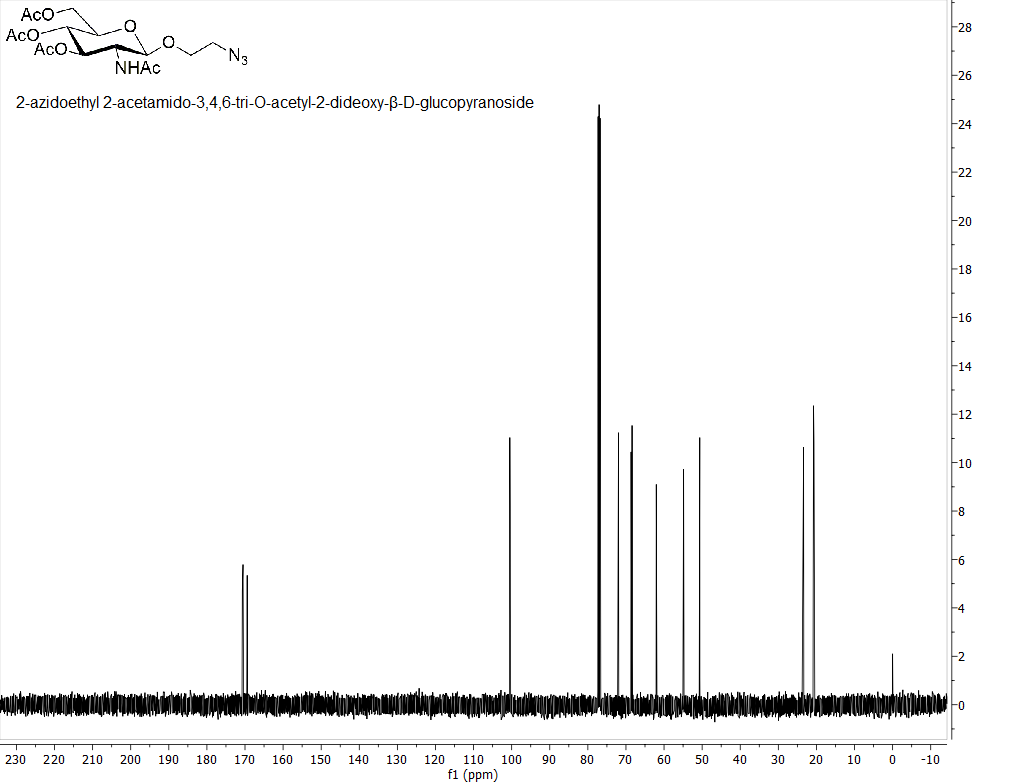
**

**Figure S15. Carbon NMR in CDCl_3_ of 2-azidoethyl 2-acetamido-3,4,6-tri-*O*-acetyl-2-dideoxy-*β*-D-glucopyranoside (2).**

**
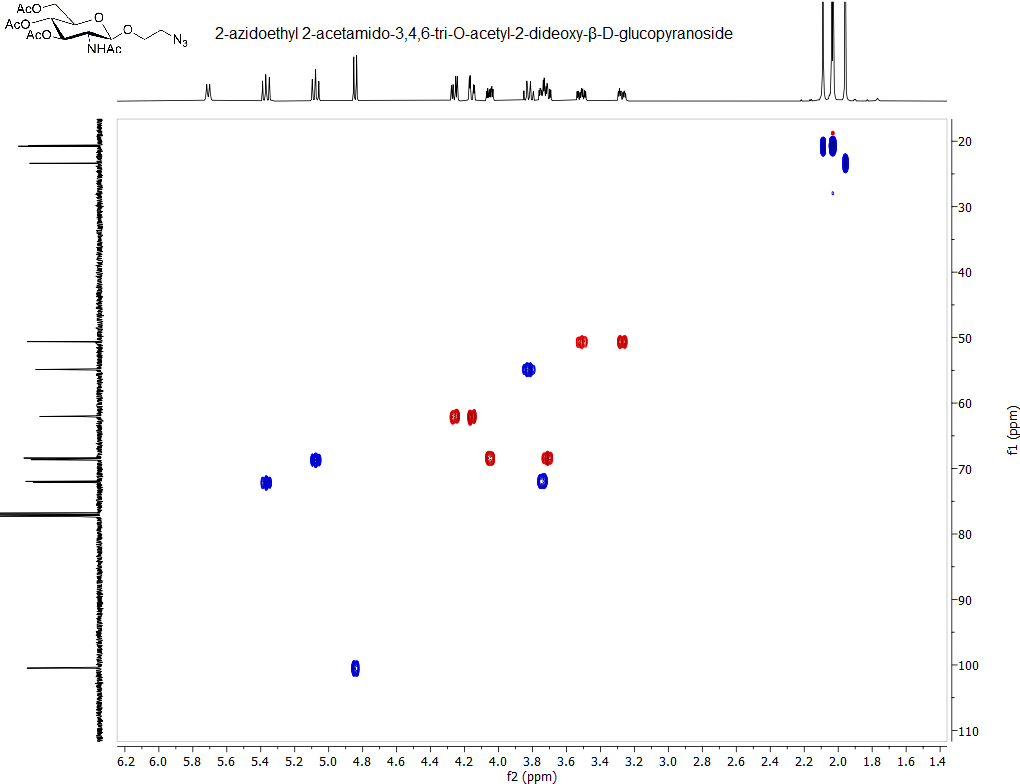
**

**Figure S16. ^1^H-^13^C-HSQC NMR in CDCl_3_ of 2-azidoethyl 2-acetamido-3,4,6-tri-*O*-acetyl-2-dideoxy-*β*-D-glucopyranoside (2).**

**
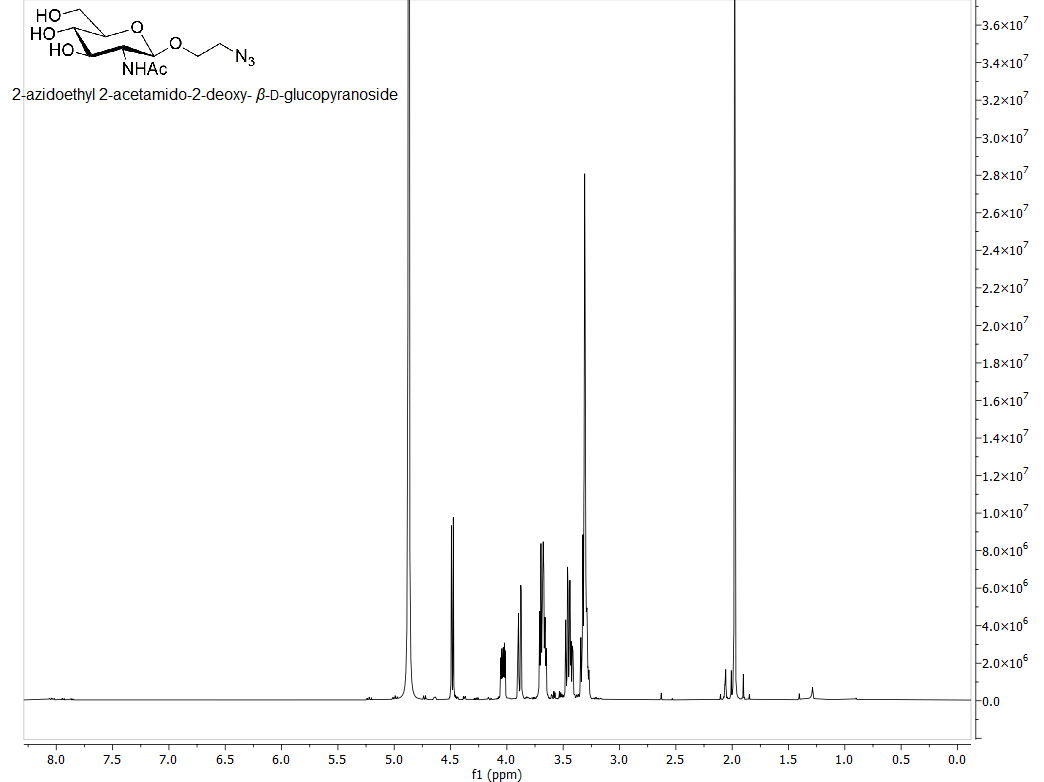
**

**Figure S17. Proton NMR in CD_3_OD of 2-azidoethyl 2-acetamido-2-deoxy-*β*-D-glucopyranoside (3).**

**
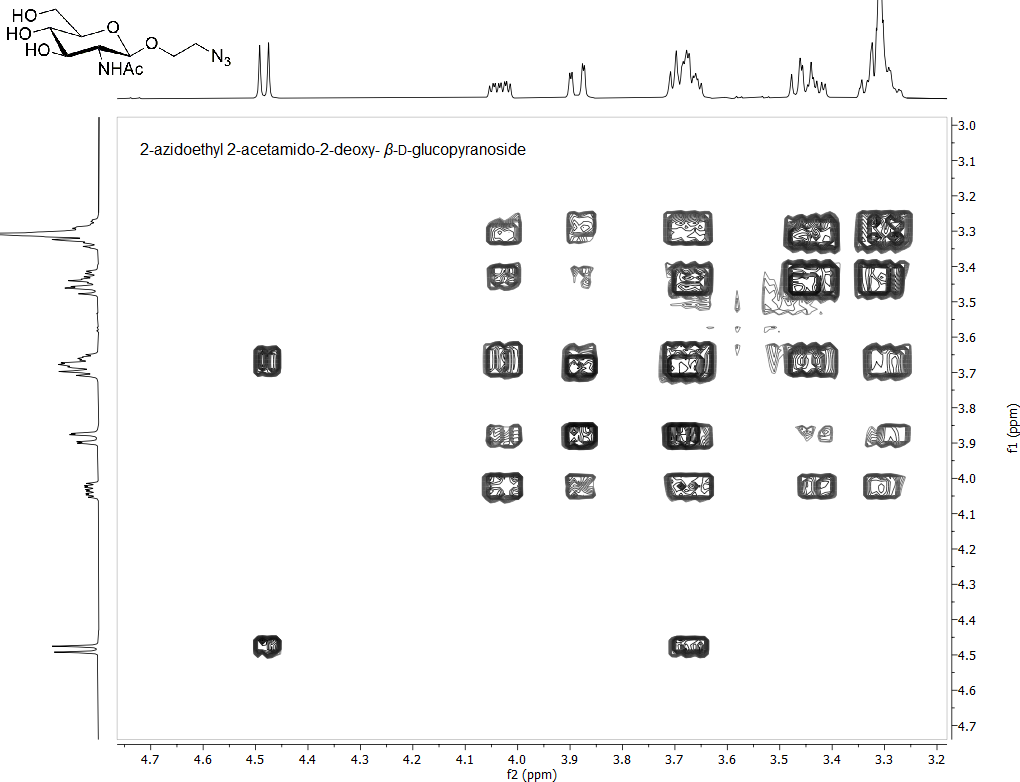
**

**Figure S18. ^1^H-^1^H-COSY NMR in CD_3_OD of 2-azidoethyl 2-acetamido-2-deoxy-*β*-D-glucopyranoside (3).**

**
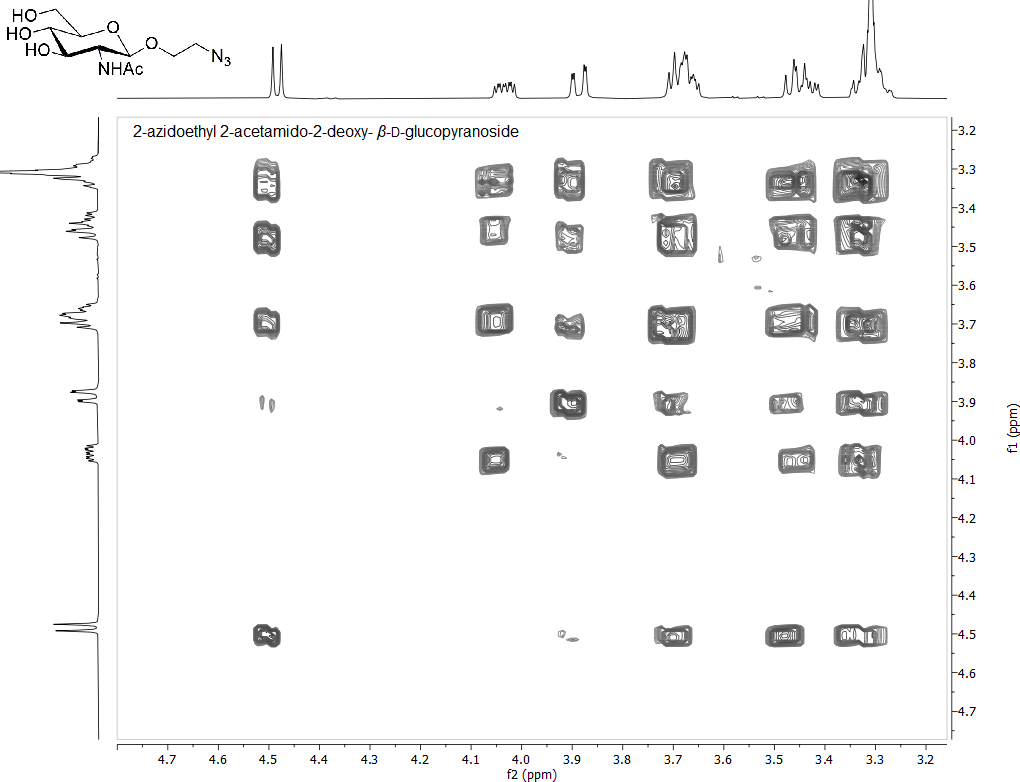
**

**Figure S18. ^1^H-^1^H-TOCSY NMR in CD_3_OD of 2-azidoethyl 2-acetamido-2-deoxy-*β*-D-glucopyranoside (3).**

**
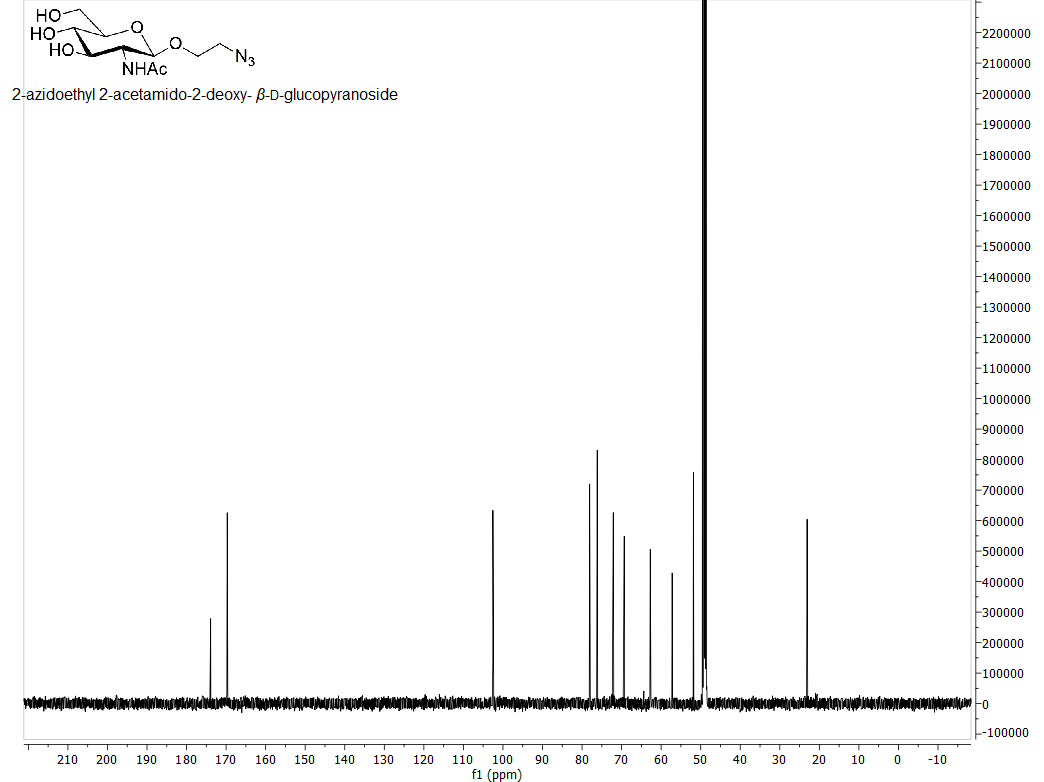
**

**Figure S19. Carbon NMR in CD_3_OD of 2-azidoethyl 2-acetamido-2-deoxy-*β*-D-glucopyranoside (3).**

**
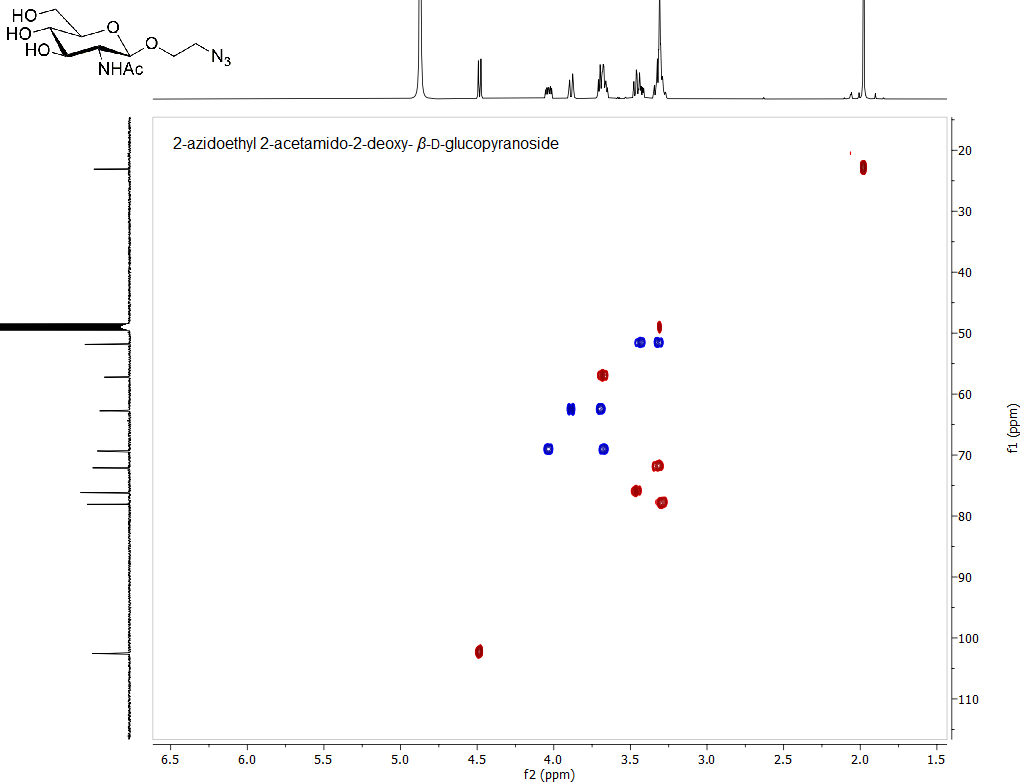
**

**Figure S20. ^1^H-^13^C-HSQC NMR in CD_3_OD of 2-azidoethyl 2-acetamido-2-deoxy-*β*-D-glucopyranoside (3).**

**
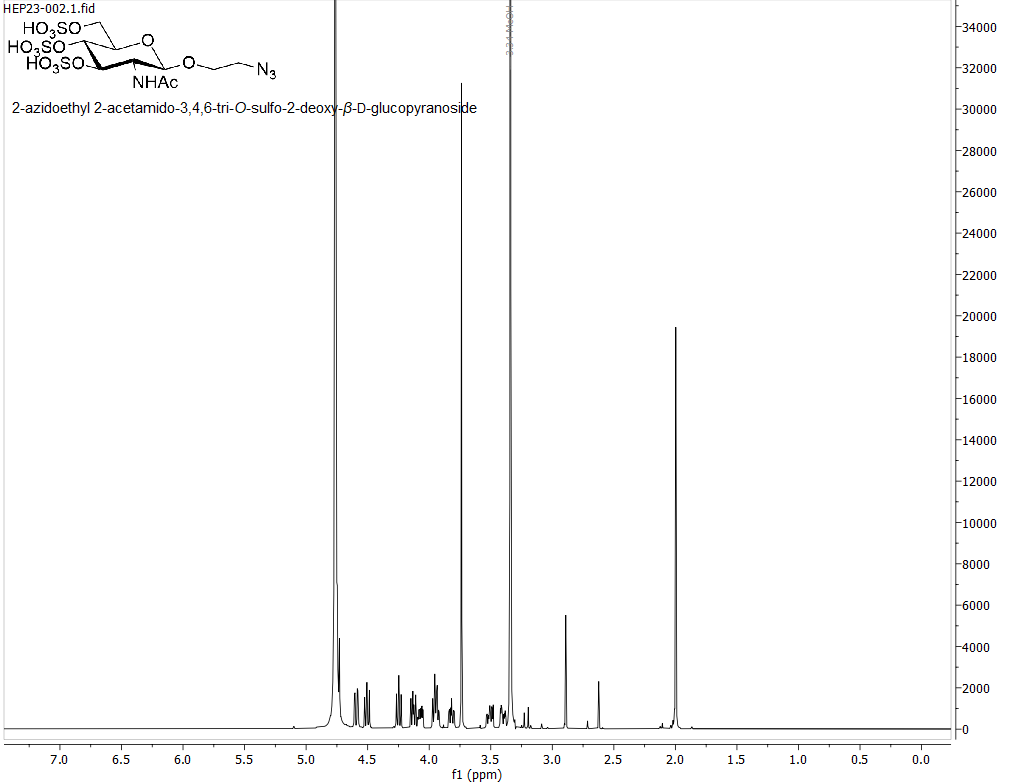
**

**Figure S21. Proton NMR in D_2_O of 2-azidoethyl 2-acetamido-3,4,6-tri-*O*-sulfo-2-deoxy-*β*-D-glucopyranoside (4).**

**
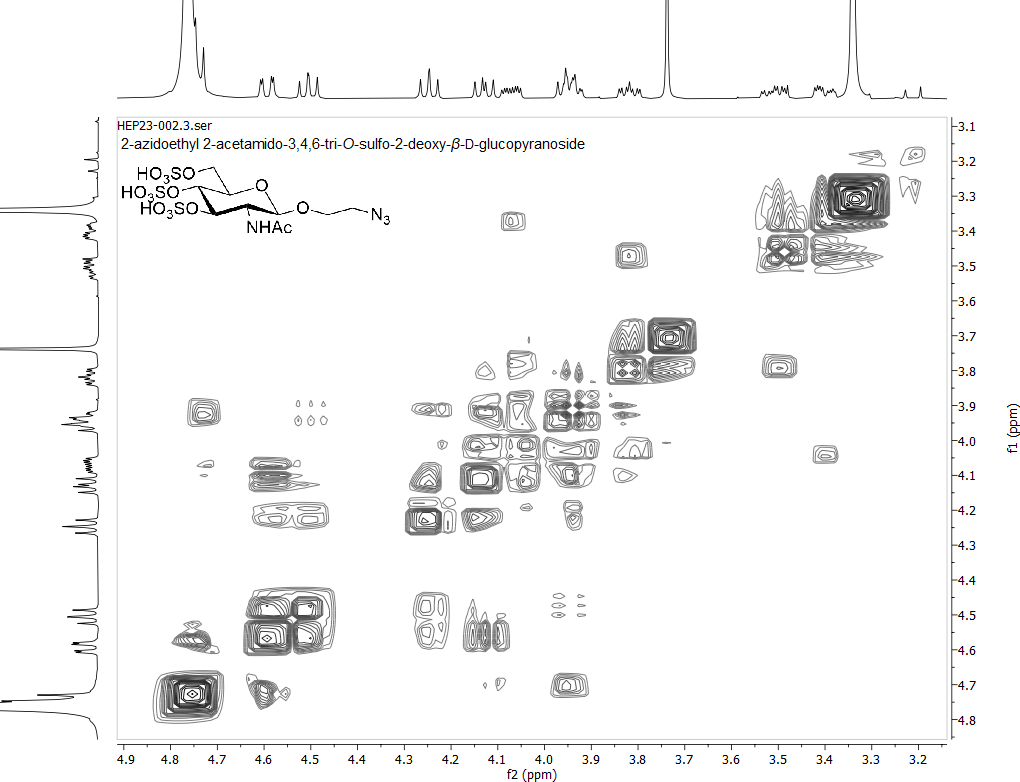
**

**Figure S22. ^1^H-^1^H-COSY NMR in D_2_O of 2-azidoethyl 2-acetamido-3,4,6-tri-*O*-sulfo-2-deoxy-*β*-D-glucopyranoside (4).**

**
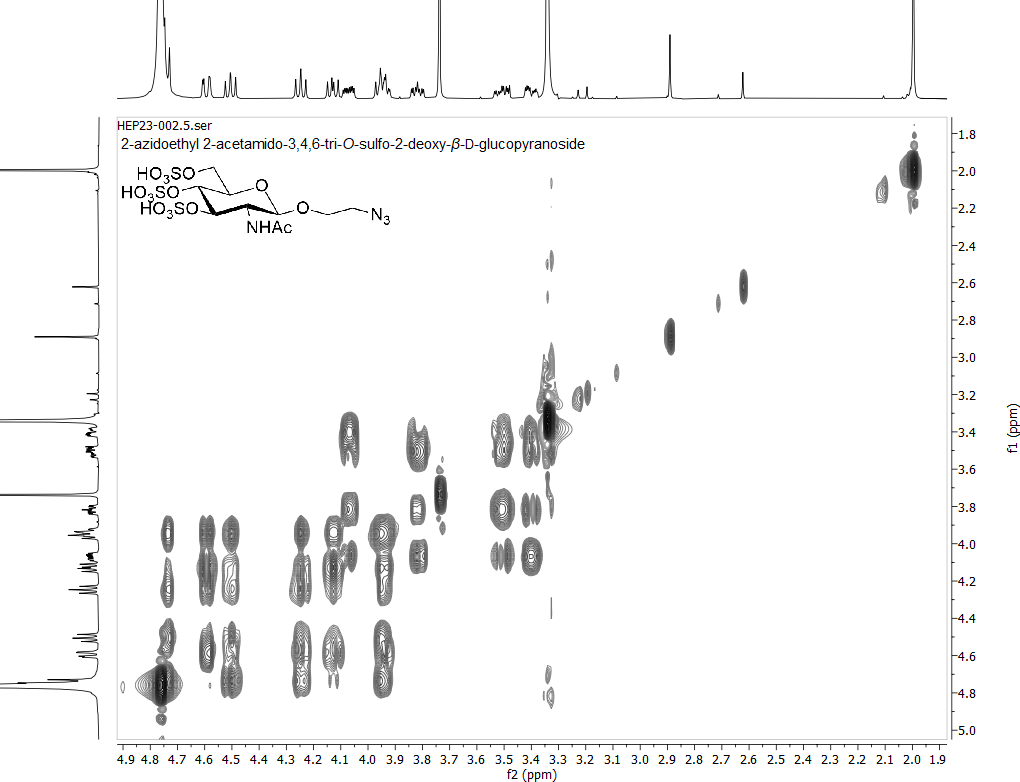
**

**Figure S23. ^1^H-^1^H-TOCSY NMR in D_2_O of 2-azidoethyl 2-acetamido-3,4,6-tri-*O*-sulfo-2-deoxy-*β*-D-glucopyranoside (4).**

**
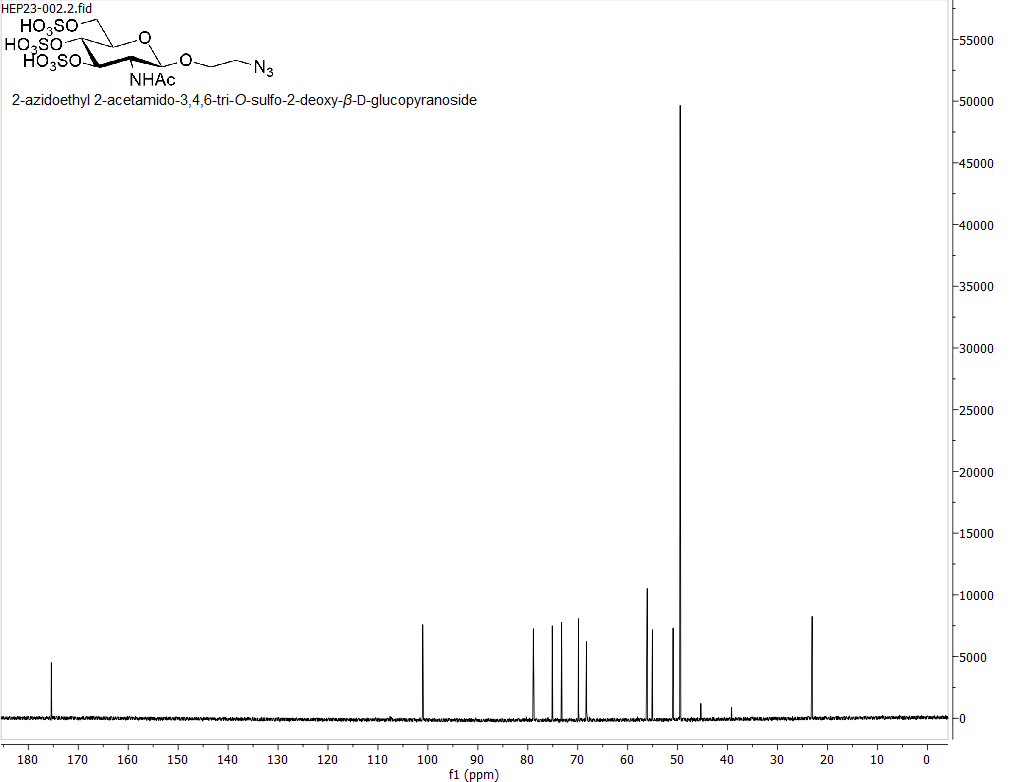
**

**Figure S24. Carbon NMR in D_2_O of 2-azidoethyl 2-acetamido-3,4,6-tri-*O*-sulfo-2-deoxy-*β*-D-glucopyranoside (4).**

**
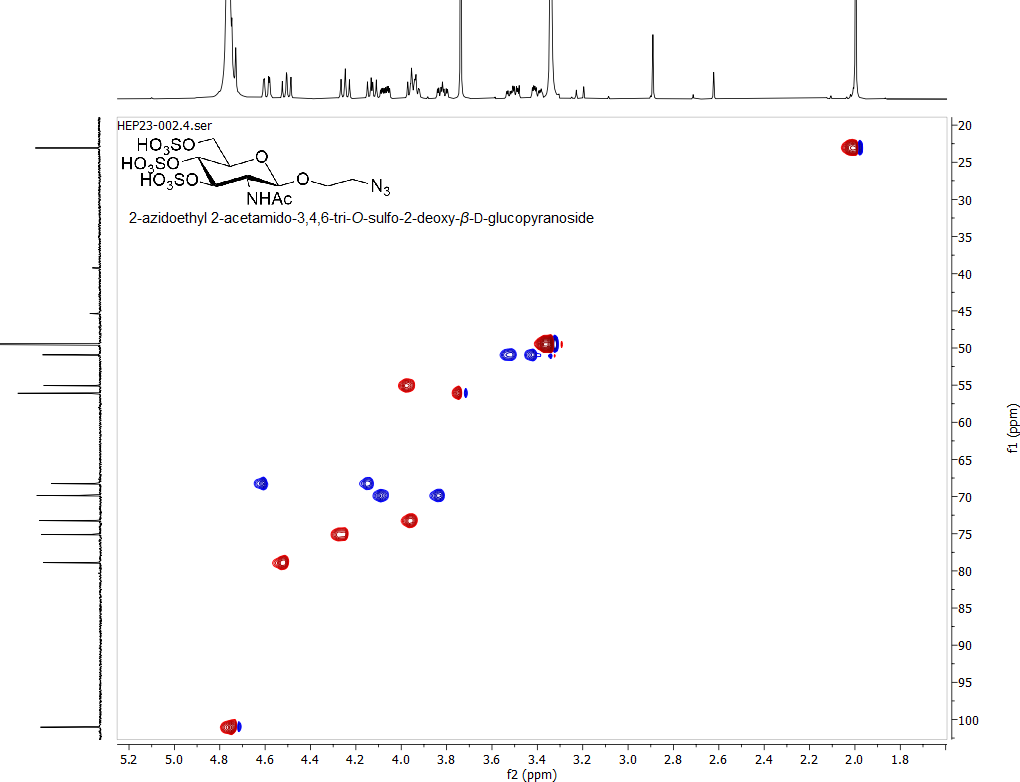
**

**Figure S25. ^1^H-^13^C-HSQC NMR in D_2_O of 2-azidoethyl 2-acetamido-3,4,6-tri-*O*-sulfo-2-deoxy-*β*-D-glucopyranoside (4).**


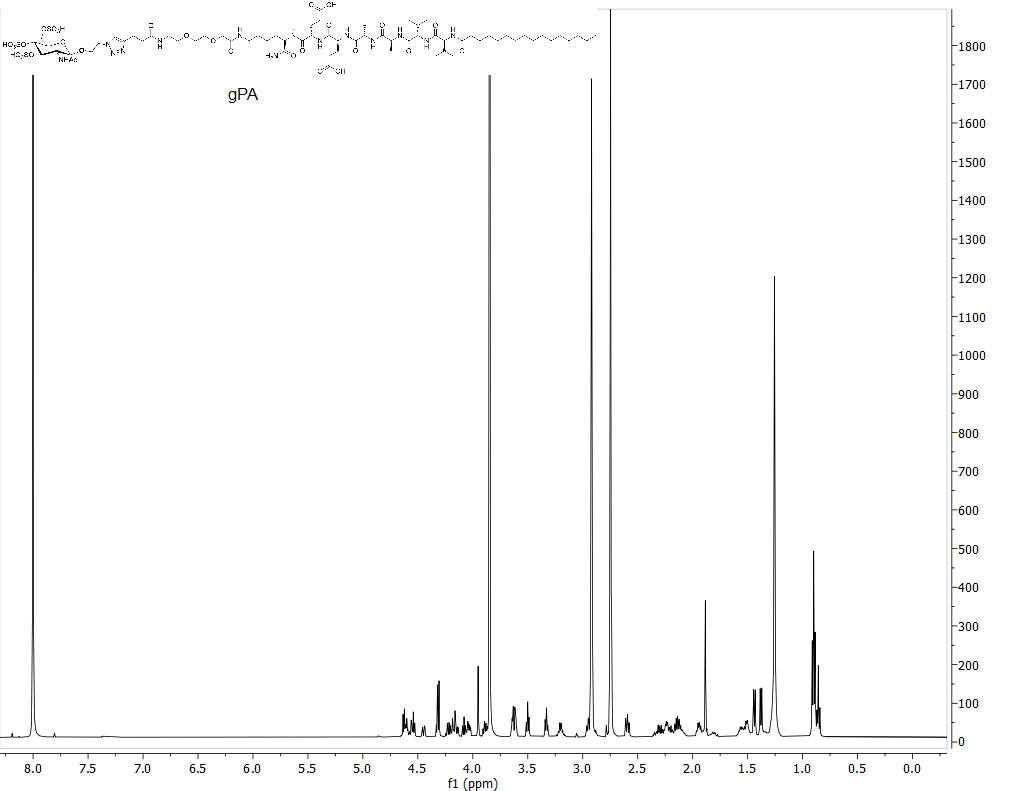


**Figure S26. Proton NMR in DMF-d_7_ of gPA.**

**
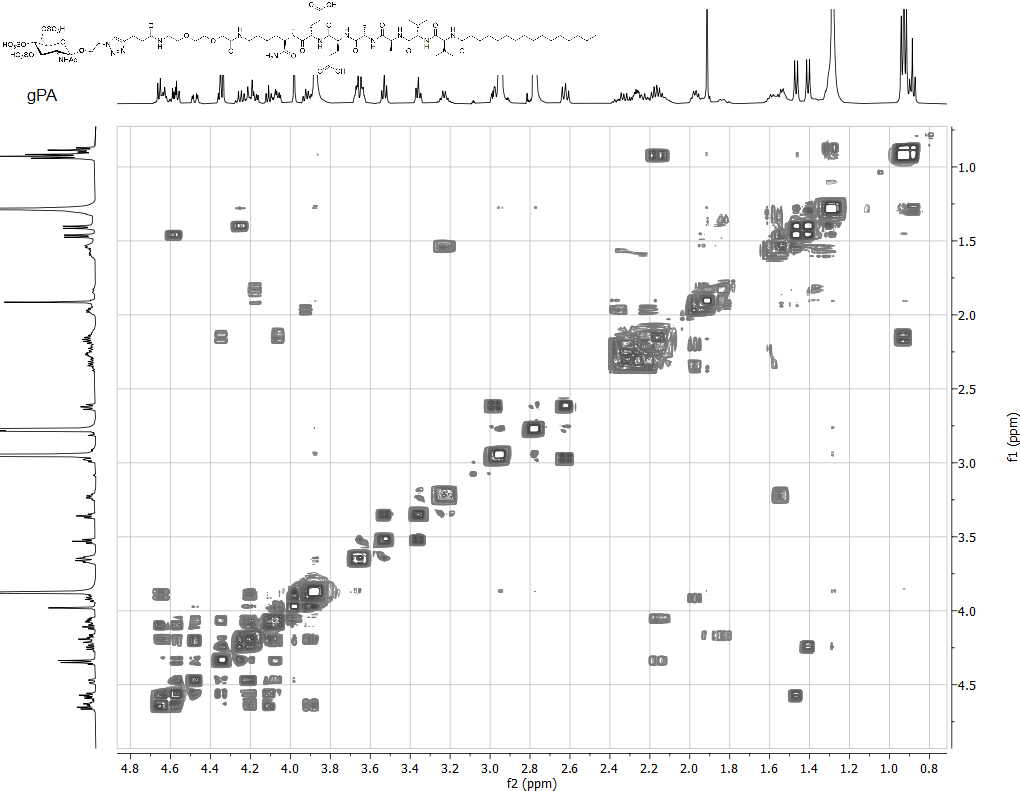
**

**Figure S27. ^1^H-^1^H-COSY NMR in DMF-d_7_ of gPA.**

**
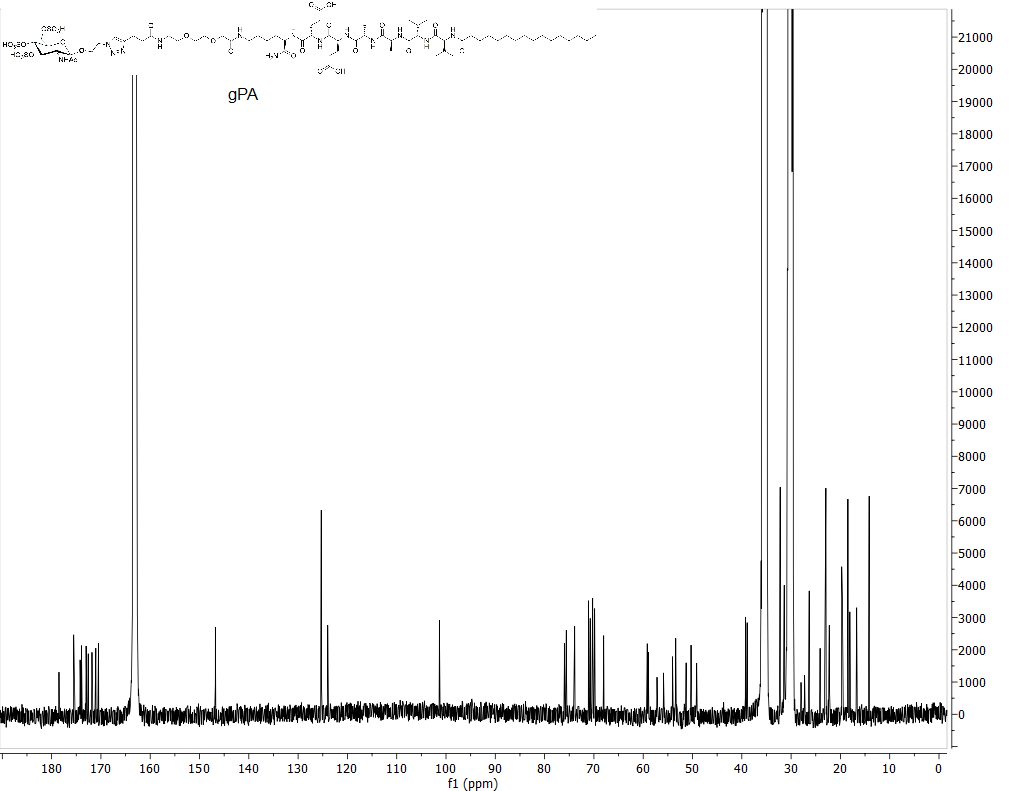
**

**Figure S28. Carbon NMR in DMF-d_7_ of gPA.**

**
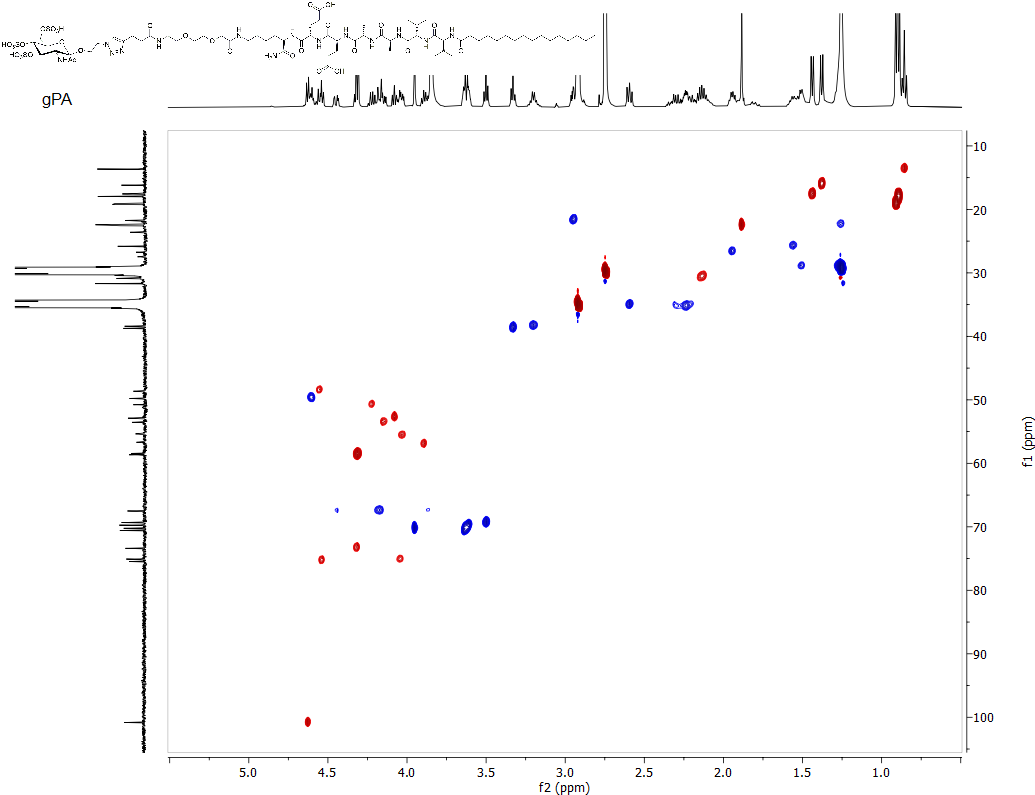
**

**Figure S29. ^1^H-^13^C-HSQC NMR in DMF-d_7_ of gPA.**

**
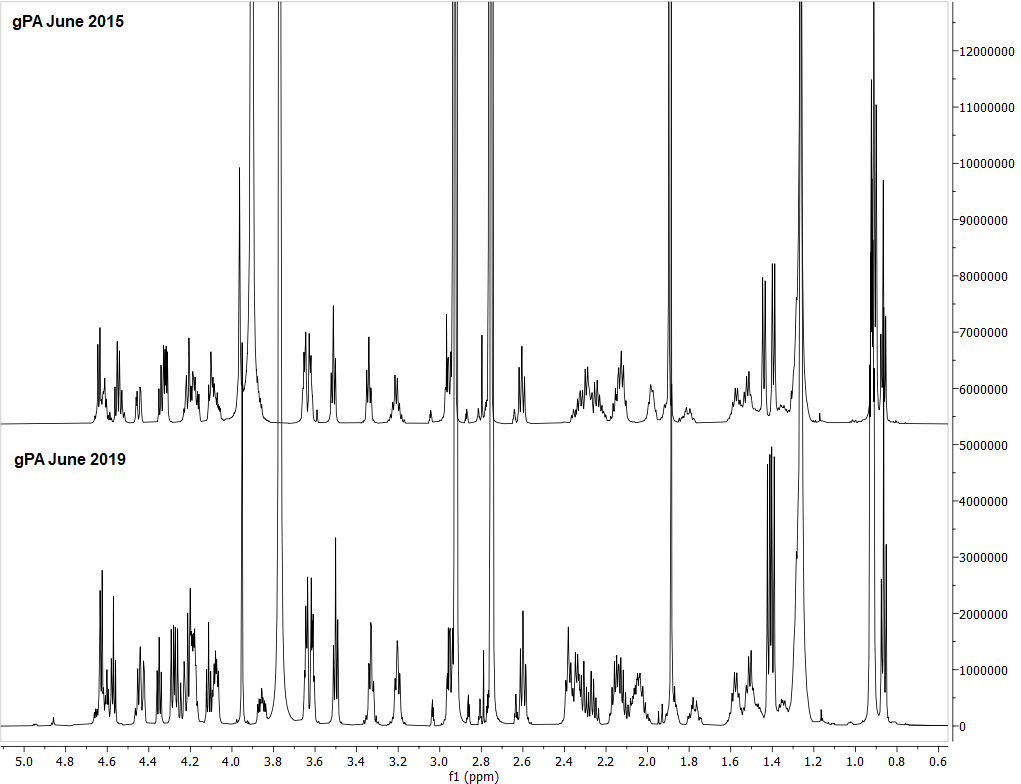
**

**Figure S30. Comparative NMR spectra recorded in DMF-d_7_ on June 2015 and June 2019 using the same batch of gPA to test stability.**

**
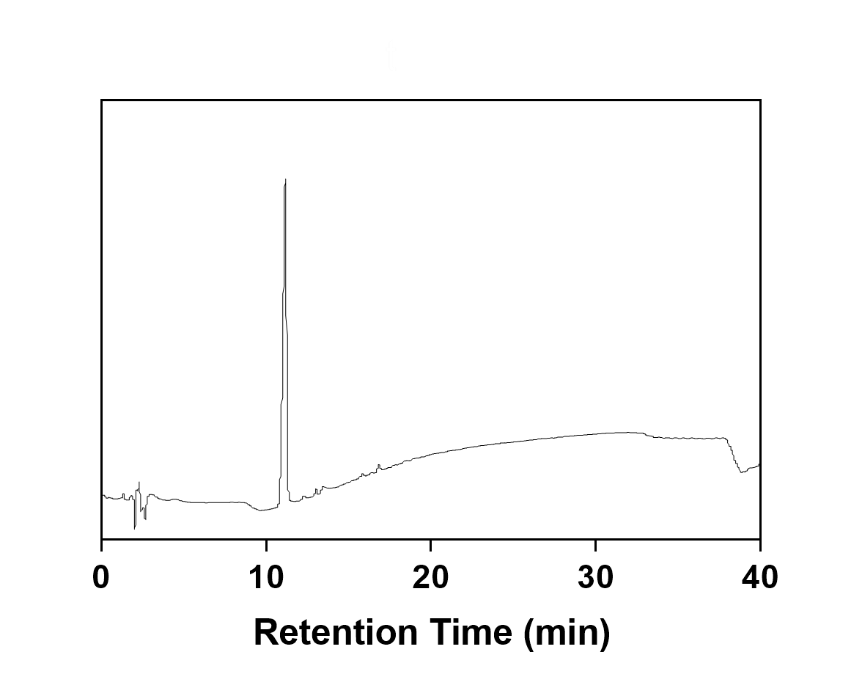
**

**Figure S31. LCMS trace of gPA, [gPA] = 1 mg/ml. Recorded in December 2019.**

**
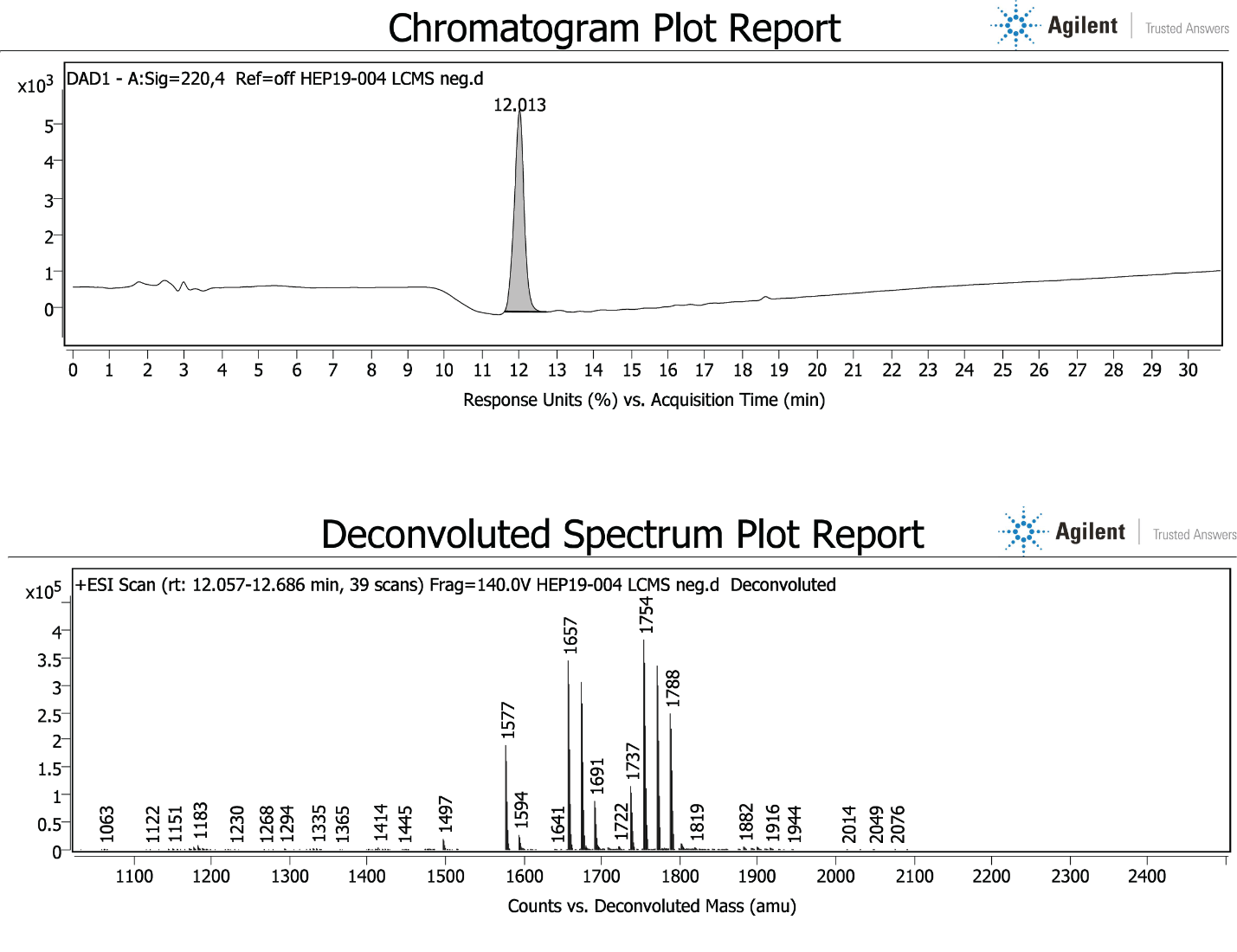
**

**Figure S32. LCMS analysis recorded in December 2023, testing the storage stability over time, using the same batch of gPA as analyzed in December 2019 (See S31).**
